# Total synthesis of justicidin B, justicidin E, and taiwanin C: A general and flexible approach toward the synthesis of natural arylnaphthalene lactone lignans

**DOI:** 10.3389/fchem.2022.1103554

**Published:** 2022-12-22

**Authors:** Kai Wei, Yucui Sun, Yiren Xu, Wen Hu, Ying Ma, Yi Lu, Wen Chen, Hongbin Zhang

**Affiliations:** ^1^ Key Laboratory of Medicinal Chemistry for Natural Resource, Ministry of Education, Yunnan Provincial Center for Research and Development of Natural Products, Yunnan Characteristic Plant Extraction Laboratory, School of Pharmacy, Yunnan University, Kunming, China; ^2^ Henan Engineering Research Center of Funiu Mountain’s Medical Resources Utilization and Molecular Medicine, School of Medical Sciences, Pingdingshan University, Pingdingshan, China

**Keywords:** total synthesis, natural products, arylnaphthalene lactone lignans, Suzuki cross-coupling, cation-induced cyclization

## Abstract

Lignans are widely present in traditional medicinal plants. Many natural arylnaphthalene lactone lignans (NALLs) isolated from the genera *Justicia*, *Haplophyllum*, and *Phyllanthus* possess interesting biological activities. Herein, we report a general strategy for the total synthesis of this kind of lignans. Features of this new approach are an aryl–alkyl Suzuki cross-coupling to introduce the dioxinone unit, a cation-induced cyclization to construct the aryl dihydronaphthalene, and base-mediated oxidative aromatization to furnish the arylnaphthalene core. By incorporating these key transformations, the total syntheses of justicidins B and E and taiwanin C covered type I and type II NALLs were accomplished.

## 1 Introduction

**FIGURE 1:**
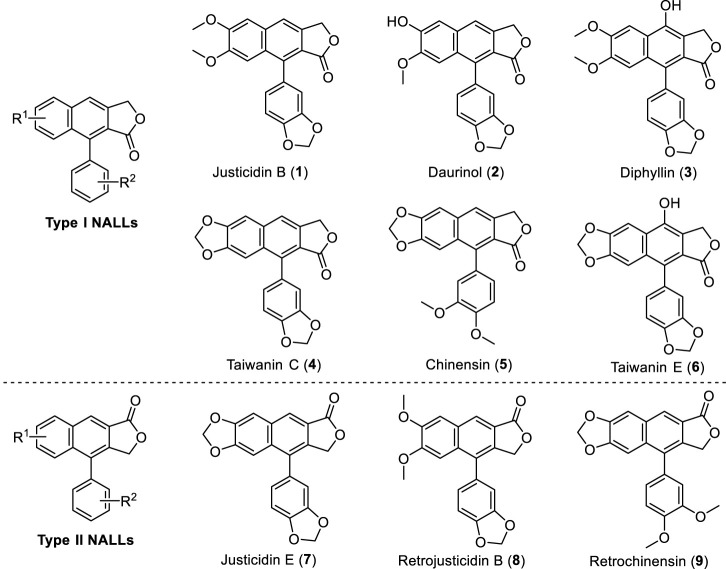
Representative NALLs.

Natural arylnaphthalene lactone lignans (NALLs) are widely isolated from the plant family *Acanthaceae* (Day et al., 1999; Shen et al., 2004; Zhang et al., 2007; Jin et al., 2014; Jiang et al., 2017; Jin et al., 2017; Lv et al., 2021; Liu et al., 2022), Euphorbiaceae (Anjaneyulu et al., 1981; Wu et al., 2006) and Rutaceae (Gözler et al., 1984; Sheriha et al., 1984; Hesse et al., 1992; Ulubelen et al., 1994; Gözler et al., 1996), especially from the genera *Justicia*, *Haplophyllum*, and *Phyllanthus*. Many of these lignans possess a broad range of biological activities, including antimicrobial (Kawazoe et al., 2001), antifungal (Ashraf et al., 1995), anti-cancer (Wang et al., 2019), antiplatelet (Chen et al., 1996; Weng et al., 2004), antiprotozoal (Gertsch et al., 2003), antimetastatic (Hajdu et al., 2014), antiviral (Sagar et al., 2004; Yeo et al., 2005; Janmanchi et al., 2010), cytotoxic (Day et al., 2002; Chang et al., 2003; Susplugas et al., 2005; Vasilev et al., 2006), and neuroprotective activities (Chun et al., 2017) in cell-based assays or animal models. For instance, justicidin B exhibits powerful antimicrobial activity (El-Gendy et al., 2008) and inhibitory activity against the Sindbis virus (Charlton, 1998). Meanwhile, taiwanin C exhibits important antiplatelet activity (Daron et al., 2022) and was found to be a potent COX inhibitor (Ban et al., 2002). Some representative natural arylnaphthalene lactone lignans (**1**–**9**) are shown in Figure 1.

Because of their important pharmacological properties, NALLs have attracted attention from the organic synthetic community since the pioneering synthetic work on these lignans in 1895 by Michael et al. (1895). Synthetic efforts have resulted in many impressive approaches toward these highly substituted 1-arylnaphthalenes and culminated in the total synthesis of a series of arylnaphthalene lactone-type lignans (Chen et al., 2018; Zhao et al., 2018; Park et al., 2020). Methodologies for the construction of 1-arylnaphthalenes could be roughly classified into five categories: Diels–Alder type cycloaddition (Brown et al., 1964; Holmes et al., 1971; Klemm et al., 1971; Takano et al., 1985; Stevenson et al., 1989; Padwa et al., 1996; Xiong et al., 2012; Kudoh et al., 2013; Kocsis et al., 2014; Park et al., 2014; Meng et al., 2016), benzannulation (Ogiku et al., 1995; Flanagan et al., 2002; Nishii et al., 2005; Ishikawa et al., 2021; Moriguchi et al., 2021), Garratt–Braverman-type cyclization (Mondal et al., 2011; Mondal et al., 2012), transition metal-mediated cyclization (Murakami et al., 1998; Mizufune et al., 2001; Sato et al., 2004; Sato et al., 2007; Gudla et al., 2011; Patel et al., 2013; Wong et al., 2014; Kao et al., 2015; Naresh et al., 2015; Xiao et al., 2018), and other type of annulations (Ogiku et al., 1990; Kamal et al., 1994; Ogiku et al., 1995; Harrowven et al., 2001; Foley et al., 2010; He et al., 2014; Hayat et al., 2015; Yamamoto et al., 2015).

Inspired by these well-designed processes and our previous efforts on cation-induced cyclization (Chen et al., 2017; Chen et al., 2019; Wei et al., 2021; Chen et al., 2022; Li et al., 2022), we recently developed an intramolecular cation-induced reaction to synthesize the highly substituted 1-aryl dihydronaphthalene unit, an advanced precursor of natural arylnaphthalene lactone lignans. In this paper, we report a general and flexible strategy toward the synthesis of justicidin E (type II NALLs), justicidin B, and taiwanin C (type I NALLs) based on this efficient cation-induced cyclization.

## 2 Results and discussion

### 2.1 Retrosynthetic analysis

**SCHEME 1:**
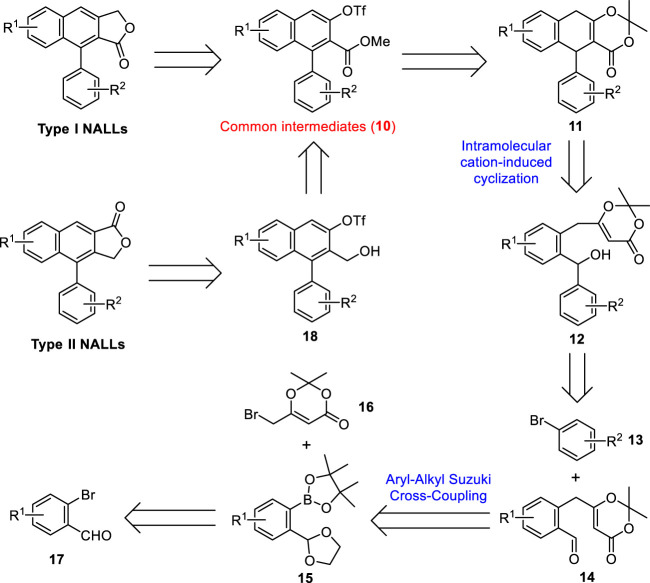
Retrosynthetic analysis for both type I and II NALLs.

Our retrosynthetic analysis for both type I and type II NALLs is shown in Scheme 1. Type I NALLs could be achieved by a Stille cross-coupling between common intermediates (**10**) and tributylstannyl methanol followed by lactonization (Zhang et al., 2019). Type II NALLs could be accessed *via* carbonylative lactonization (Crisp et al., 1995) of triflate **18**, which could be obtained *via* a reduction from common intermediates (**10**). Ring opening of dioxinone **11** followed by subsequent base-mediated oxidation (Zhao et al., 2020) and triflation would lead to methyl ester **10**. Dihydronaphthalene **11** could be accessed through the intramolecular cation-induced cyclization of alcohol **12**, which could be prepared by a selective nucleophilic addition of aryl lithium generated *in situ* from aryl bromide **13** to aldehyde **14**. Aldehyde **14** was expected to be formed by an aryl–alkyl Suzuki cross-coupling between pinacolyl borate **15** and commercially available alkyl bromide **16** followed by a deprotection of the ketal moiety. Borate **15** could be obtained from commercially available bromide **17** *via* functional group protection, halogen–lithium exchange reaction, and borylation.

### 2.2 Total synthesis of justicidin B

**SCHEME 2:**
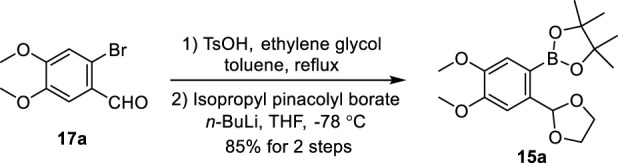
Gram-scale synthesis of pinacolyl borate **15a**. Bu: butyl, THF: tetrahydrofuran, and Ts: *p*-toluenesulfonyl.

We chose justicidin B, a type I NALL, as the first target of our synthetic journey. Our synthesis began with the preparation of pinacolyl borate **15a** (Scheme 2). Treatment of commercially available bromo-aldehyde **17a** with ethylene glycol provided its acetal, after subsequent halogen–lithium exchange by exposing it with *n*-butyllithium followed by borylation (Nagaki et al., 2012) provided **15a** in 85% yield.

With pinacolyl borate in hand, we next explored aryl–alkyl Suzuki cross-coupling between borate **15a** and commercially available alkyl bromide **16** (Table 1). Although numerous conditions for Suzuki cross-coupling reactions between alkyl halide and aryl boric acid or borate have been developed, using alkyl bromide **16** as a coupling partner to accomplish this cross-coupling reaction is still challenging due to the thermosensitive and base-sensitive dioxinone unit present in substrate **16** (Reber et al., 2009; Katsuki et al., 2017).

**TABLE 1:** Optimization for the aryl–alkyl Suzuki cross-coupling^a^.

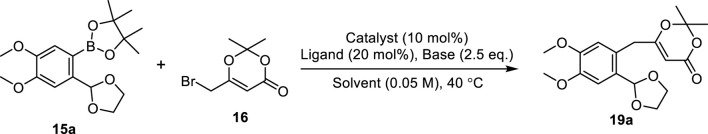					
Entry	Catalyst	Ligand	Base	Solvent	Yield [%]^b^
1	Pd(PPh_3_)_4_	-	K_3_PO_4_	1,4-Dioxane	Trace
2	Pd(OAc)_2_	PPh_3_	K_3_PO_4_	1,4-Dioxane	2
3	Pd(dppf)Cl_2_	PPh_3_	K_3_PO_4_	1,4-Dioxane	3
4	Pd_2_(dba)_3_	PPh_3_	K_3_PO_4_	1,4-Dioxane	4
5	Pd(dba)_2_	PPh_3_	K_3_PO_4_	1,4-Dioxane	8
6	Pd(dba)_2_	PPh_3_	K_2_CO_3_	1,4-Dioxane	3
7	Pd(dba)_2_	PPh_3_	Na_2_CO_3_	1,4-Dioxane	0
8	Pd(dba)_2_	PPh_3_	Cs_2_CO_3_	1,4-Dioxane	5
9	Pd(dba)_2_	PPh_3_	KOAc	1,4-Dioxane	2
10	Pd(dba)_2_	*t*-Bu_3_P	K_3_PO_4_	1,4-Dioxane	20
11	Pd(dba)_2_	PCy_3_	K_3_PO_4_	1,4-Dioxane	26
12	Pd(dba)_2_	X-Phos	K_3_PO_4_	1,4-Dioxane	Trace
13	Pd(dba)_2_	S-Phos	K_3_PO_4_	1,4-Dioxane	51
14	Pd(dba)_2_	S-Phos	K_3_PO_4_	DMF	Trace
15	Pd(dba)_2_	S-Phos	K_3_PO_4_	THF	71
16	Pd(dba)_2_	S-Phos	K_3_PO_4_	CPME	51
17	Pd(dba)_2_	S-Phos	K_3_PO_4_	TBME	63
18	Pd(dba)_2_	S-Phos	K_3_PO_4_	DME	77

^a^ The reactions were performed with **15a** (0.2 mmol), **16** (0.26 mmol), catalyst (10 mol%), ligand (20 mol%), base (2.5 eq.), and solvent (3 ml) at 40°C for 7 h.

^b^ Yields represent isolated yields. Ac: acetyl, Bu: butyl, CPME: cyclopentyl methyl ether, Cy: cyclohexyl, dba: dibenzylideneacetone, DME: 1,2-dimethoxyethane, DMF: *N*,*N*-dimethylformamide, dppf: 1,1′-*bis*(diphenylphosphino)ferrocene, Ph: phenyl, S-Phos 2-dicyclohexylphosphino-2′,6′-dimethoxybiphenyl, TBME: *tert*-butyl methyl ether, X-Phos 2-(dicyclohexylphosphino)-2′,4′,6′-tri-*i*-propyl-1,1′-biphenyl.

In order to optimize the yield of this cross-coupling reaction, a systematic screening of reaction conditions was conducted (Table 1). Initially, we used the regular catalyst Pd(PPh_3_)_4_ employed in Suzuki cross-coupling (Miyaura et al., 1979). Not surprisingly, Pd(PPh_3_)_4_ was completely ineffective for the desired cross-coupling (Table 1, entry 1). Reactions were then conducted at a 0.2-mmol scale with several commercially available palladium catalysts (10 mol%) in the presence of PPh_3_ (20 mol%) and K_3_PO_4_ in 1,4-dioxane (Table 1, entries 2–5). We found that Pd(dba)_2_ served as an efficient Pd source for this coupling process (Table 1, entry 5). Next, the bases were screened, and the yield of the desired product **19a** was not increased with a number of bases (Table 1, entries 5–9). A number of ligands were then used. We found that a ligand has a significant impact on the efficiency of this cross-coupling reaction (Table 1, entries 9–13). When the S-Phos ligand was used, the desired product **19a** could be obtained with 51% yield (Table 1, entry 9). With the catalytic system in hand, we next screened the solvents, and DME gave the best results (Table 1, entries 13–18). Finally, the optimum reaction conditions for this coupling reaction (Table 1, entry 18) were established.

**SCHEME 3:**
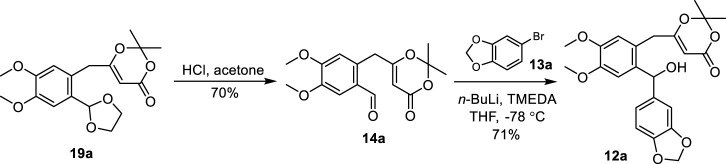
Synthesis of benzhydrol **12a**. TMEDA: *N*,*N*,*N′*,*N′*-tetramethylethylenediamine.

Next, the acetal protecting group of compound **19a** was removed with HCl in acetone to produce aldehyde **14a** (Scheme 3). The treatment of **13a** with *n*-BuLi followed by the addition of aldehyde **14a** unfortunately failed to yield the desired benzhydrol **12a**. To promote the desired reaction, a number of additives were used including hexamethylphosphoric acid triamide (HMPA), *N*,*N*-dimethyl propylene urea (DMPU), and *N*,*N*,*N′*,*N′*-tetramethylethylenediamine (TMEDA). The addition of TMEDA provided benzhydrol **12a** at 71% yield.

With benzhydrol **12a** in hand, we next focused on the proposed cation-induced cyclization (Table 2). A number of Brønsted acids and Lewis acids (Table 2, entries 1–6) were used. Although the cyclization could be promoted by Brønsted acids, BF_3_·Et_2_O provided the best yield (Table 2, entry 6). The yield of the targeted product could be further improved when the reaction was conducted at a lower temperature (Table 2, entry 8). This cation-induced cyclization could be scaled up to 2.1 mmol (Table 2, entry 8, 0.90 g, and 68% yield).

**TABLE 2:** Optimization for the intramolecular cation-induced cyclization^a^.

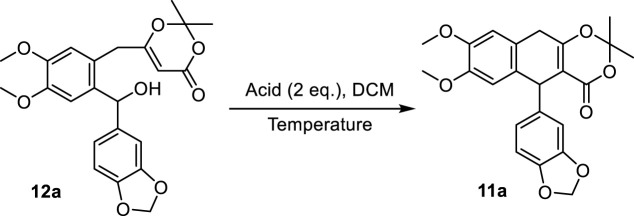			
Entry	Acid	Temperature [^o^C]	Yield [%]^b^
1	TfOH	0	Trace
2	TFA	0	40
3	CSA	0	19
4	TsOH	0	43
5	TMSCl	0	46
6	BF_3_·Et_2_O	0	50
7	BF_3_·Et_2_O	-30	60
8	BF_3_·Et_2_O	-40	69
9	BF_3_·Et_2_O	-50	61
10	BF_3_·Et_2_O	-40	68^c^

^a^ The reactions were performed with **12a** (0.2 mmol), acid (2.0 eq.), and solvent (3 ml) for 3 h.

^b^ Yields represent isolated yields.

^c^ The reaction was conducted at a 2.1-mmol scale. CSA: camphorsufonic acid, DCM: dichloromethane, Et: ethyl, and TFA: trifluoroacetic acid.

**SCHEME 4:**
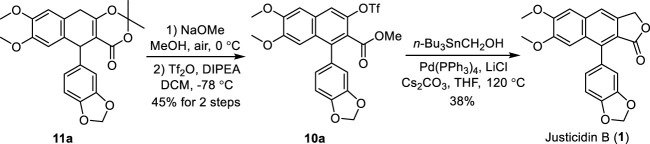
Total synthesis of justicidin B (**1**). DIPEA: diisopropylethylamine, Me: methyl.

Having established the procedure for advanced intermediate **11a**, research focus was then directed toward the total synthesis of justicidin B **1**). The treatment of **11a** with sodium methoxide in MeOH under air followed by the addition of Tf_2_O and DIPEA in DCM produced the first common intermediate **10a** in 45% yield (Scheme 4). It is noteworthy that an oxidative (by air) aromatization occurred under strong basic conditions. Next, a Pd-catalyzed Stille cross-coupling of triflate **10a** with tributylstannyl methanol in the presence of Pd(PPh_3_)_4_, Cs_2_CO_3_, and LiCl followed by spontaneous lactonization provided natural justicidin B (Zhang et al., 2019). The NMR spectra of our synthetic sample were in full agreement with those reported in the literature (Okigawa et al., 1970; Borges et al., 2018).

### 2.3 Total synthesis of taiwanin C and justicidin E

**SCHEME 5:**
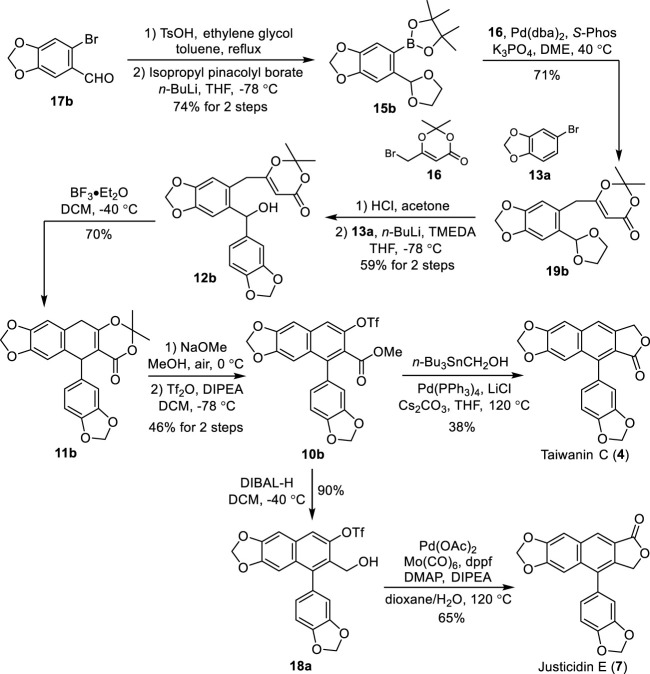
Total synthesis of taiwanin C (**4**) and justicidin E (7). DMAP: 4-dimethylaminopyridine, dppf: 1,1′-*bis*(diphenylphosphino)ferrocene.

To demonstrate the generality and flexibility of our strategy, the total syntheses of naturally occurring arylnaphthalene lignans taiwanin C (type I) and justicidin E (type II) were conducted accordingly. Treatment of commercially available piperonyl bromide **17b** with ethylene glycol in the presence of TsOH followed by a halogen–lithium exchange and borylation afforded the pinacolyl borate **15b** in 74% yield (Scheme 5). Suzuki cross-coupling of bromide **16** with **15b** under the optimum reaction conditions afforded the corresponding dioxinone **19b**. Deprotection of the acetal of **19b** with HCl in acetone followed by a selective 1,2-addition with the 3,4-methylenedioxyphenyllithium, which was generated *in situ* from the halogen–lithium exchange between bromide **13a** and *n*-BuLi, yielded the benzhydrol **12b** in 59% for two steps.

Aryl dihydronaphthalene **11b** was obtained successfully in 70% yield through our intramolecular cation-induced cyclization from benzhydrol **12b**. The treatment of **11b** with NaOMe in MeOH under air followed by triflation with Tf_2_O afforded the common intermediate **10b** in 46% yield for two steps. Reaction of **10b** with tributylstannyl methanol in the presence of Pd(PPh_3_)_4_, Cs_2_CO_3_, and LiCl produced the natural taiwanin C **4**). Reduction of **10b** with DIBAL-H provided the alcohol **18a** in 90% yield. Natural justicidin E (7) was furnished in 38% isolated yield *via* an improved Pd-catalyzed carbonylative lactonization of triflate **18a** with Co(CO)_6_. The NMR spectra of these two synthetic samples agree well with the reported literature (Anjaneyulu et al., 1981; Subbaraju et al., 1996; Flanagan et al., 2002).

## 3 Conclusion

We have developed a general and flexible strategy for the synthesis of justicidin B, taiwanin C, and justicidin E from commercially available materials. Key transformations to the success of the synthesis were an aryl–alkyl Suzuki cross-coupling, an intramolecular cation-induced cyclization, and a base-mediated oxidative aromatization. Our new approach paves the way toward the synthesis of biologically active natural arylnaphthalene lactone lignans and could be used for the preparation of their analogues.

## Data Availability

The raw data supporting the conclusions of this article will be made available by the authors, without undue reservation.
