# Supplementary material for: Total synthesis of justicidin B, justicidin E, and taiwanin C: A general and flexible approach toward the synthesis of natural arylnaphthalene lactone lignans
Source: Front Chem. 2022 Dec 22;10:1103554. doi: 10.3389/fchem.2022.1103554 (PMC9815507; doi:10.3389/fchem.2022.1103554)
Supplement: Supplementary file 1 [file DataSheet1.PDF]

A Flexible Approach towards Synthesis of Natural Arylnaphthalene Lactone Lignans Justicidin B, Justicidin E and Taiwanin C

Kai Wei[1,2†], Yucui Sun[1†], Yiren Xu[1†], Wen Hu[1], Ying Ma[1], Yi Lu[1], Wen Chen[1*], Hongbin Zhang[1*]

[1]*Key Laboratory of Medicinal Chemistry for Natural Resource, Ministry of Education; Yunnan Provincial Center for Research & Development of Natural Products; Yunnan Characteristic Plant Extraction Laboratory; School of Pharmacy, Yunnan University, Kunming 650500, P. R. China*

[2]*Henan Engineering Research Center of Funiu Mountain's Medical Resources Utilization and Molecular Medicine, School of Medical Sciences, Pingdingshan University, Pingdingshan, P. R. China*

*** Correspondence:**
Wen Chen: wenchen@ynu.edu.cn
Hongbin Zhang: zhanghb@ynu.edu.cn

[†] These authors have contributed equally to this work.

Table of Contents

1 General information 3

2 Experimental procedures and data for synthetic intermediates ...3

2.1 Pinacolyl borate **15a** 3

2.2 Optimization for the aryl-alkyl Suzuki cross-coupling 5

2.3 Dioxinone **19a** 6

2.4 Aldehyde **14a** 7

2.5 Benzhydrol **12a** 7

2.6 Dihydronaphthalene **11a** 8

2.7 Methyl Ester **S1** 9

2.8 Triflate **10a** 10

2.9 Justicidin B (**1**) 11

2.10 Pinacolyl borate **15b** 12

2.11 Dioxinone **19b** 13

2.12 Aldehyde **14b** 14

2.13 Benzhydrol **12b** 14

2.14 Dihydronaphthalene **11b** 15

2.15 Triflate **10b** 16

2.16 Taiwanin C (**4**) 17

2.17 Alcohol **18a** 18

2.18 Justicidin E (**7**) 19

3 Copies of ^{1}H and ^{13}C NMR Spectra 21

1 General information

^{1}H-NMR and ^{13}C-NMR spectra were recorded on Bruker Avance 400 and 600 spectrometers. Chemical shifts are reported in parts per million (δ) referenced to tetramethylsilane (0.0 ppm), chloroform (7.26 ppm or 77.16 ppm) and methanol (3.31 ppm or 49.0 ppm), respectively. Data for ^{1}H-NMR and ^{13}C-NMR spectroscopy are reported as follows: chemical shift (δ ppm), multiplicity (s = singlet, d = doublet, t = triplet, q = quartet, m = multiplet, br = broad), coupling constant (Hz), and integration. High Resolution Mass spectra were taken on AB QSTAR Pulsar mass spectrometer or Aglient LC/MSD TOF mass spectrometer. All new compounds were characterized by ^{1}H NMR, ^{13}C NMR and HRMS. Silica gel (200–300 mesh) for column chromatography and silica GF_{254} for TLC were obtained from Merck Chemicals Co. Ltd. (Shanghai). Anhydrous THF, CPME, TBME, and DME were dried by distillation over metallic sodium and benzophenone. Anhydrous dichloromethane, toluene and methanol were distilled from calcium hydride. Starting materials and reagents used in reactions were obtained commercially from Acros, Aldrich and Adamas-beta®, and were used without purification, unless otherwise indicated. All reactions were conducted in dried glassware under a positive pressure of dry nitrogen or argon.

2 Experimental procedures and data for synthetic intermediates

2.1 Pinacolyl borate 15a

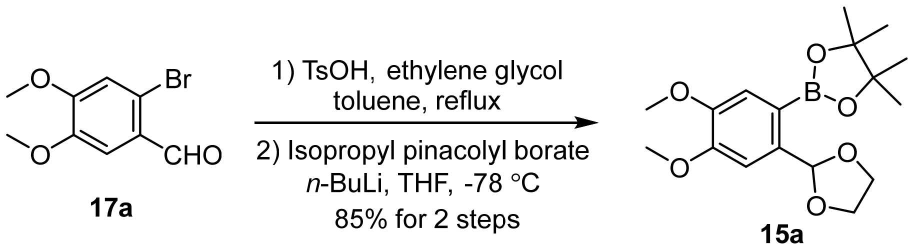

17a

15a

To a mixture of 6-bromoveratraldehyde **17a** (7.35 g, 30.0 mmol) and ethylene glycol (16.9 mL, 300 mmol) in benzene (80 mL) was added *p*-TsOH·H_2O (516.2 mg, 3.0 mmol). The reaction mixture was refluxed under a Dean-Stark trap for 5 h and then cooled to room temperature. The reaction mixture was then quenched carefully with saturated *aq.* $NaHCO_3$ (20 mL) at 0 °C before being diluted with water (200 mL). The aqueous solution was extracted with EtOAc (3 × 150 mL). The combined organic phases were washed with brine (150 mL), dried over anhydrous Na_2SO_4, filtered and concentrated under reduced pressure to obtain the crude acetal which was used for the next step without further purification. The crude acetal was dissolved in dry THF (150 mL), and *n*-BuLi (1.6 M, 17.1 mL, 28.5 mmol) was then added drop by drop via a syringe at –78 °C. After being stirred at –78 °C for 30 min, isopropyl pinacolyl borate (8.7 mL, 72.8 mmol) was added drop by drop to the reaction mixture via a syringe at the same temperature. After TLC analysis, saturated *aq.* NH_4Cl (80 mL) was added carefully to the reaction mixture and the resulting mixture was extracted with EtOAc (3 × 150 mL), and the combined organic phases were dried over anhydrous Na_2SO_4. After removal of the solvents under reduced pressure, the residue was purified by flash column chromatography on silica gel (petroleum ether/EtOAc = 10:1) to afford pinacolyl borate **15a** (8.56 g, 85% for 2 steps) as a white solid. Caution: This unstable pinacolyl borate should be used immediately for the next step.

HRMS (ESI): Calcd for $C_{17}H_{25}BO_6$ $[M+H]^+$: 337.1817, found: 337.1811.

2.2 Optimization for the aryl-alkyl Suzuki cross-coupling

Table S1 Optimization for the aryl-alkyl Suzuki cross-coupling[a]

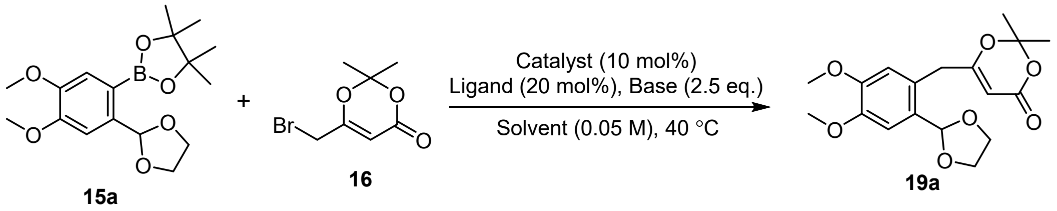

Entry	Catalyst	Ligand	Base	Solvent	Yield [%][b]
1	$Pd(PPh_3)_4$	-	K_3PO_4	1,4-dioxane	trace
2	$Pd(OAc)_2$	PPh_3	K_3PO_4	1,4-dioxane	2
3	$Pd(dppf)Cl_2$	PPh_3	K_3PO_4	1,4-dioxane	3
4	$Pd_2(dba)_3$	PPh_3	K_3PO_4	1,4-dioxane	4
5	$Pd(dba)_2$	PPh_3	K_3PO_4	1,4-dioxane	8
6	$Pd(dba)_2$	PPh_3	K_2CO_3	1,4-dioxane	3
7	$Pd(dba)_2$	PPh_3	Na_2CO_3	1,4-dioxane	0
8	$Pd(dba)_2$	PPh_3	Cs_2CO_3	1,4-dioxane	5
9	$Pd(dba)_2$	PPh_3	KOAc	1,4-dioxane	2
10	$Pd(dba)_2$	*t*-Bu_3P	K_3PO_4	1,4-dioxane	20
11	$Pd(dba)_2$	PCy_3	K_3PO_4	1,4-dioxane	26
12	$Pd(dba)_2$	X-Phos	K_3PO_4	1,4-dioxane	trace
13	$Pd(dba)_2$	S-Phos	K_3PO_4	1,4-dioxane	51
14	$Pd(dba)_2$	S-Phos	K_3PO_4	DMF	trace
15	$Pd(dba)_2$	S-Phos	K_3PO_4	THF	71
16	$Pd(dba)_2$	S-Phos	K_3PO_4	CPME	51
17	$Pd(dba)_2$	S-Phos	K_3PO_4	TBME	63
18	$Pd(dba)_2$	S-Phos	K_3PO_4	DME	77

[a]The reactions were performed with **15a** (0.2 mmol), **16** (0.26 mmol), catalyst (10 mol%), ligand (20 mol%), base (2.5 eq.) and solvent (3 mL) at 40 °C for 7 h. [b]Yields represent isolated yields. Ac acetyl, Bu butyl, CPME cyclopentyl methyl ether, Cy cyclohexyl, dba dibenzylideneacetone, DME 1,2-dimethoxyethane, DMF *N*,*N*-dimethylformamide, dppf 1,1'-*bis*(diphenylphosphino)ferrocene, Ph phenyl, S-Phos 2-dicyclohexylphosphino-2',6'-dimethoxybiphenyl, TBME *tert*-butyl methyl ether, X-Phos 2-(dicyclohexylphosphino)-2',4',6'-tri-*i*-propyl-1,1'-biphenyl.

2.3 Dioxinone 19a

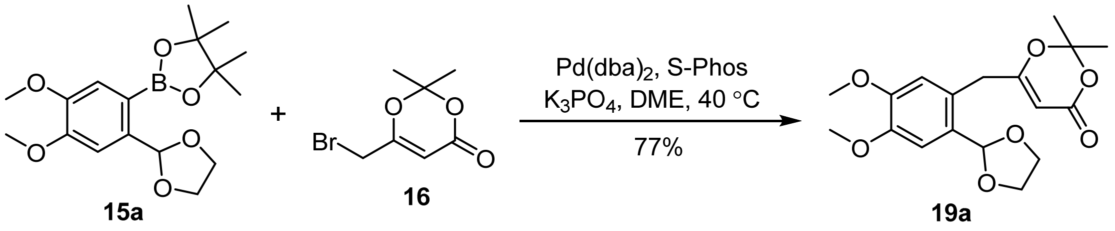

To a solution of pinacolyl borate **15a** (69.1 mg, 0.20 mmol) and alkyl bromide **16** (57.2 mg, 0.26 mmol) in DME (3 mL) was added Pd(dba)$_2$ (12.0 mg, 0.02 mmol), S-Phos (16.1 mg, 0.04 mmol) and K_3PO_4 (115.2 mg, 0.5 mmol) at room temperature under Ar. After being stirred at 40 °C for 7 h, the resulting mixture was filtered through a short column of silica gel and washed with ethyl acetate (ca. 15 mL). The organic layer was concentrated under reduced pressure. The residue was purified by column chromatography on silica gel (petroleum ether/EtOAc = 4:1) to afford dioxinone **19a** (54.3 mg, 77%) as a pale-yellow solid.

^{1}H NMR (400 MHz, CDCl$_3$) δ ppm 7.10 (s, 1H), 6.66 (s, 1H), 5.83 (s, 1H), 5.09 (s, 1H), 4.14-4.11 (m, 2H), 4.04-3.98 (m, 2H), 3.89 (s, 3H), 3.86 (s, 3H), 3.61 (s, 2H), 1.63 (s, 6H).

^{13}C NMR (100 MHz, CDCl$_3$) δ ppm 170.7, 161.5, 149.5, 148.3, 128.4, 125.0, 113.8, 109.9, 106.7, 101.7, 94.3, 65.2, 56.2, 56.1, 36.2, 25.1.

HRMS (ESI): Calcd for $C_{18}H_{22}O_7$ [M+Na]$^+$: 373.1258, found: 373.1253.

2.4 Aldehyde 14a

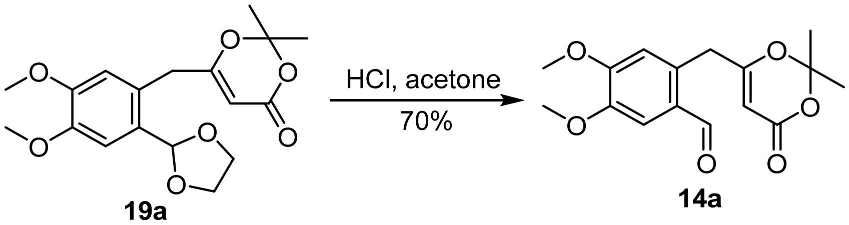

To a solution of dioxinone **19a** (700.2 mg, 2.0 mmol) in acetone (20 mL) was added HCl (2 N, 3.0 mL, 6.0 mmol) at room temperature. The reation mixture was allowed to stir at room temperature for 24 h. After TLC analysis, the reaction was quenched with saturated *aq*. $NaHCO_3$ (10 mL). The resulting mixture was then diluted with water (10 mL), and extracted with EtOAc (3 × 10 mL). The combined organic phases were washed with brine (20 mL), dried over anhydrous Na_2SO_4, filtered, and concentrated under reduced pressure. The residue was purified by column chromatography on silica gel (petroleum ether/EtOAc = 4:1) to give aldehyde **14a** (428.3 mg, 70%) as a white solid.

^{1}H NMR (400 MHz, $CDCl_3$) δ ppm 10.01 (s, 1H), 7.33 (s, 1H), 6.73 (s, 1H), 4.98 (s, 1H), 3.94 (s, 3H), 3.94 (s, 3H), 3.90 (s, 2H), 1.63 (s, 6H).

^{13}C NMR (100 MHz, $CDCl_3$) δ ppm 190.2, 170.2, 161.2, 153.7, 148.7, 130.3, 127.4, 114.6, 114.4, 107.00, 94.1, 56.4, 56.3, 36.2, 25.1.

HRMS (ESI): Calcd for $C_{16}H_{18}O_6$ $[M+Na]^+$: 329.0996, found: 329.0990.

2.5 Benzhydrol 12a

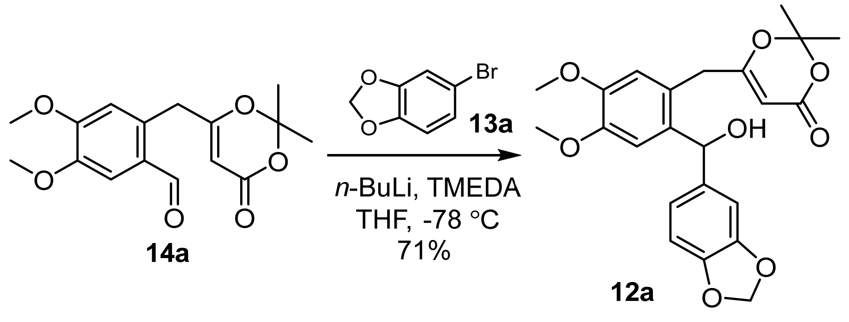

To a solution of aryl bromide **13a** (1.47 g, 7.53 mmol) and TMEDA (853.1 mg, 7.53 mmol) in dry THF (30 mL) was added *n*-BuLi (2.5 M, 2.8 mL 7.0 mmol) at -78 ºC under Ar. The mixture stirred at -78 ºC for 30 min. A solution of aldehyde **14a** (450.3 mg, 1.47 mmol) in THF (10 mL) was then added dropwise and the mixture allowed to stir for 3 h. The mixture was quenched with saturated *aq.* NH_4Cl (5 mL). The resulting mixture was then diluted with water (10 mL), and extracted with EtOAc (3 × 20 mL). The combined organic phases were washed with brine (30 mL), dried over anhydrous Na_2SO_4, filtered, and concentrated under reduced pressure. The residue was purified by column chromatography on silica gel (petroleum ether/EtOAc = 4:1) to give benzhydrol **12a** (472.3 mg, 75%) as a pale-yellow oil.

^{1}H NMR (400 MHz, $CDCl_3$) δ ppm 7.07 (s, 1H), 6.73 (s, 2H), 6.72 (s, 1H), 6.63 (s, 1H), 5.91 (d, J = 0.8 Hz, 2H), 4.92 (s, 1H), 3.86 (s, 3H), 3.85 (s, 3H), 3.43 (s, 2H), 1.59 (s, 6H)

^{13}C NMR (100 MHz, $CDCl_3$) δ ppm 170.5, 161.2, 148.6, 148.5, 148.0, 147.3, 137.1, 134.6, 123.5, 120.5, 114.00, 110.9, 108.3, 107.6, 106.7, 101.3, 94.2, 73.0, 56.2, 56.1, 36.2, 25.1, 25.0.

HRMS (ESI): Calcd for $C_{23}H_{24}O_8$ $[M+Na]^+$: 451.1363, found: 453.1360.

2.6 Dihydronaphthalene 11a

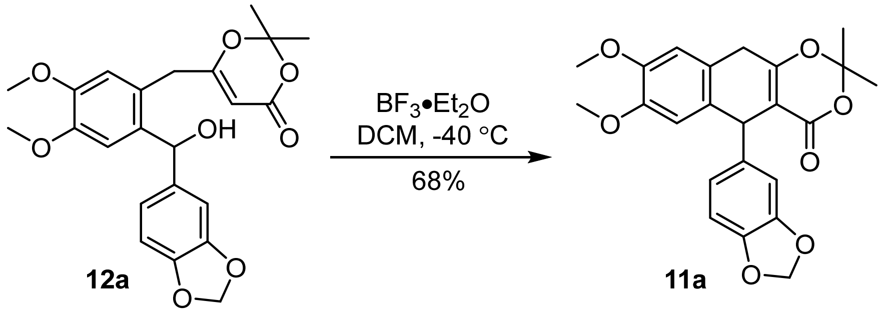

The benzhydrol **12a** (900.0 mg, 2.1 mmol) was dissolved in dry DCM (25 mL), and BF_3•Et_2O (0.53 mL, 4.2 mmol) was added at –40 ºC. After being stirred at –40 ºC for 4 h, the reaction was quenched with saturated *aq.* $NaHCO_3$ (10 mL) and diluted with

water (20 mL). The aqueous layer was then extracted with DCM (3 × 20 mL). The combined organic phases were washed with brine (30 mL) and dried over anhydrous Na_2SO_4. After removal of the solvents under reduced pressure, the residue was purified by column chromatography on silica gel (petroleum ether/EtOAc = 5:1) to yield dihydronaphthalene **11a** (585.2 mg, 68%) as a white solid.

^{1}H NMR (400 MHz, $CDCl_3$) δ ppm 6.73 (d, *J* = 1.7 Hz, 0.3H), 6.71 (d, *J* = 1.7 Hz, 0.7H), 6.69 (s, 0.7H), 6.67 (s, 0.3H), 6.63 (s, 1H), 6.59 (d, *J* = 1.6 Hz, 1H), 6.58 (s, 1H), 5.88 (d, *J* = 1.4 Hz, 1H), 5.86 (d, *J* = 1.4 Hz, 1H), 5.00 (t, *J* = 2.9 Hz, 1H), 3.86 (s, 3H), 3.76 (s, 3H), 3.74 (d, *J* = 3.4 Hz, 0.38H), 3.69 (d, *J* = 3.4 Hz, 0.62H), 3.54 (d, *J* = 2.4 Hz, 0.61H), 3.48 (d, *J* = 2.4 Hz, 0.36H), 1.69 (s, 3H), 1.59 (s, 3H).

^{13}C NMR (100 MHz, $CDCl_3$) δ ppm 162.4, 160.5, 148.4, 148.0, 147.7, 146.1, 139.0, 129.5, 121.7, 121.0, 111.6, 110.2, 108.2, 108.0, 106.0, 105.7, 100.9, 56.0, 55.9, 43.4, 31.9, 27.0, 23.8.

HRMS (ESI): Calcd for $C_{23}H_{22}O_7$ $[M+Na]^+$: 433.121258, found: 433.1257.

2.7 Methyl Ester S1

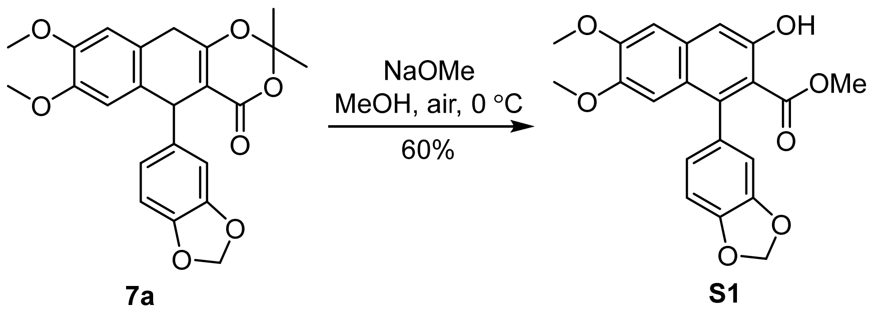

Sodium (336.1 mg, 14.6 mmol) was added portionwise to methanol (5 mL) at 0 ºC. After addition, the reaction flask was removed from the ice bath and warmed up to room temperature. After being stirred at room temperature for 30 min, compound **7a** (300.2 mg, 0.73 mmol) was introduced to the flask in one portion, and the reaction mixture was then stirred under air for 12 h. After TLC analysis, saturated *aq.* NH_4Cl (8 mL) was added to the reaction mixture at 0 ºC, and the resulting mixture was then

extracted with EtOAc (3 × 10 mL). The combined organic phases were washed with brine (30 mL), dried over anhydrous Na_2SO_4, filtered, and concentrated under reduced pressure. The residue was purified by column chromatography on silica gel (petroleum ether/EtOAc = 6:1) to give methyl ester **S1** (167.3 mg, 60%) as a pale-yellow oil.

^{1}H NMR (400 MHz, CDCl$_3$) δ ppm 10.09 (s, 1H), 7.22 (s, 1H), 6.94 (s, 1H), 6.90 (d, *J* = 7.9 Hz, 1H), 6.74 (d, *J* = 1.1 Hz, 1H), 6.70 (s, 1H), 6.67 (dd, *J* = 7.9, 1.3 Hz, 1H), 6.06 (s, 1H), 6.04 (s, 1H), 3.99 (s, 3H), 3.70 (s, 3H), 3.54 (s, 3H).

^{13}C NMR (100 MHz, CDCl$_3$) δ ppm 171.3, 154.9, 152.0, 148.0, 147.2, 146.6, 141.8, 134.1, 133.7, 123.2, 122.5, 113.1, 110.7, 109.9, 107.9, 106.4, 104.5, 101.1, 56.0, 55.6, 52.1.

HRMS (ESI): Calcd for $C_{21}H_{18}O_7$ $[M+Na]^+$: 405.0945, found: 405.0948.

2.8 Triflate 10a

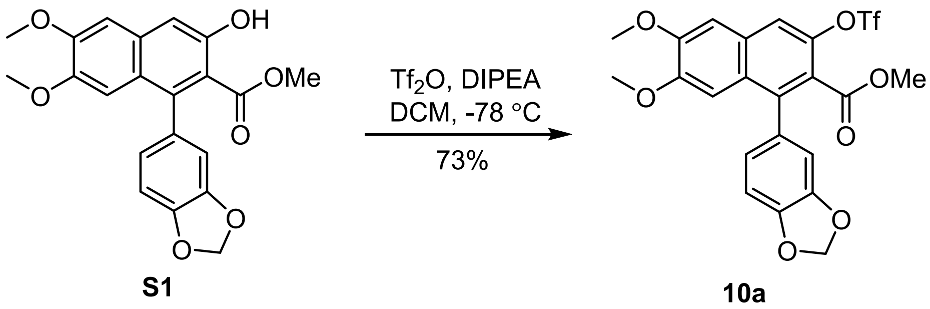

To a solution of methyl ester **S1** (200.2 mg, 0.52 mmol) in anhydrous DCM (15 mL) was added DIPEA (0.36 mL, 2.10 mmol) at –78 °C. After being stirred at –78 °C for 10 min, Tf_2O (0.18 mL, 1.05 mmol) was introduced. The reaction mixture was then stirred at –78 °C for 3 h before being quenched with saturated *aq.* $NaHCO_3$ (10 mL). The aqueous layer was extracted with DCM (3 × 10 mL), and the combined organic phases were dried over anhydrous Na_2SO_4. After removal of the solvents under reduced pressure, the residue was purified by column chromatography on silica gel (petroleum ether/EtOAc = 6:1) to give triflate **10a** (197.0 mg, 73%) which was used directly in the next reaction.

HRMS (ESI): Calcd for $C_{22}H_{17}F_3O_9S$ $[M+Na]^+$: 537.0438, found: 537.0439.

2.9 Justicidin B (1)

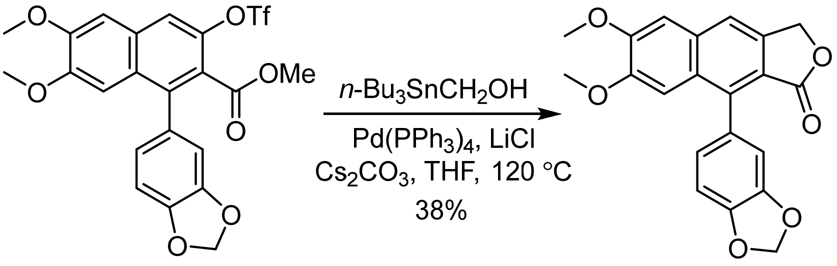

10a Justicidin B (**1**)

A flame-dried 10 mL sealed tube was sequentially charged with **10a** (51.0 mg, 0.1 mmol), LiCl (17.1 mg, 0.4 mmol), Cs_2CO_3 (65.2 mg, 0.2 mmol), tributylstannyl methanol (38.6 mg, 0.12 mmol) and Pd(PPh3)4 (11.6 mg, 0.01 mmol). And anhydrous THF (5 mL) was then added into the flask. The tube was sealed with a terflon screw top and the mixture was heated at 120 ºC under Ar for 12 h. The saturated *aq.* $NaHCO_3$ (5 mL) was then added to the reaction mixture at rt. The resulting mixture was then extracted with EtOAc (3 × 7 mL). The combined organic phases were washed with brine (10 mL), dried over Na_2SO_4, filtered, and concentrated under reduced pressure. The residue was purified by flash chromatography on silica gel (petroleum ether/EtOAc = 4:1) to afford Justicidin B (14.2 mg, 38%).

^{1}H NMR (400 MHz, $CDCl_3$) δ ppm 7.70 (s, 1H), 7.19 (s, 1H), 7.11 (s, 1H), 6.97 (d, *J* = 7.9 Hz, 1H), 6.86 (s, 1H), 6.84 (d, *J* = 7.9 Hz, 1H), 6.09 (s, 1H), 6.05 (s, 1H), 5.38 (s, 2H), 4.05 (s, 3H), 3.81 (s, 3H).

^{13}C NMR (100 MHz, $CDCl_3$) δ ppm 170.1, 152.0, 150.3, 147.7, 147.7, 139.8, 139.7, 133.3, 129.0, 128.6, 123.6, 118.7, 118.4, 110.7, 108.4, 106.2, 106.1, 101.4, 68.2, 56.2, 56.0.

HRMS (ESI): Calcd for $C_{21}H_{16}O_6$ $[M+Na]^+$: 387.0839, found: 387.0835.

2.10 Pinacolyl borate 12b

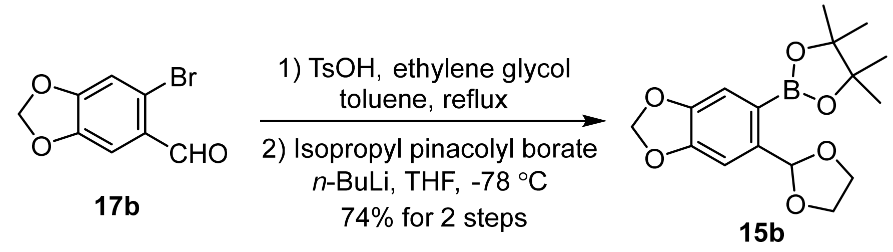

To a mixture of piperonyl bromide **17b** (9.20 g, 40.0 mmol) and ethylene glycol (11.0 mL, 200 mmol) in benzene (80 mL) was added *p*-TsOH·H_2O (691.3 mg, 4.0 mmol). The reaction mixture was refluxed under a Dean-Stark trap for 5 h and then cooled to room temperature. The reaction mixture was then quenched carefully with saturated *aq.* $NaHCO_3$ (20 mL) at 0 °C before being diluted with water (200 mL). The aqueous solution was extracted with EtOAc (3 × 150 mL). The combined organic phases were washed with brine (150 mL), dried over anhydrous Na_2SO_4, filtered and concentrated under reduced pressure to obtain the crude acetal which was used for the next step without further purification. The crude acetal was dissolved in dry THF (100 mL), and *n*-BuLi (2.5 M, 22.8 mL, 28.5 mmol) was then added drop by drop via a syringe at –78 °C. After being stirred at –78 °C for 30 min, isopropyl pinacolyl borate (11.6 mL, 57.0 mmol) was added drop by drop to the reaction mixture via a syringe at the same temperature. After TLC analysis, saturated *aq.* NH_4Cl (80 mL) was added carefully to the reaction mixture and the resulting mixture was extracted with EtOAc (3 × 150 mL), and the combined organic phases were dried over anhydrous Na_2SO_4. After removal of the solvents under reduced pressure, the residue was purified by flash column chromatography on silica gel (petroleum ether/EtOAc = 10:1) to afford pinacolyl borate **15b** (9.61 g, 75% for 2 steps) as a pale-yellow oil.

^{1}H NMR (400 MHz, $CDCl_3$) δ ppm 7.19 (s, 1H), 7.11 (s, 1H), 6.37 (s, 1H), 5.94 (s, 2H), 4.13 – 4.05 (m, 2H), 4.05 – 3.97 (m, 2H), 1.32 (s, 12H).

^{13}**C NMR (100 MHz, CDCl$_3$) δ ppm** 149.9, 147.7, 139.2, 114.2, 106.6, 101.9, 101.2, 83.8, 65.3, 24.9.

HRMS (ESI): Calcd for $C_{18}H_{21}BO_6$ $[M+Na]^+$: 343.1323, found: 343.1320.

2.11 Dioxinone 19b

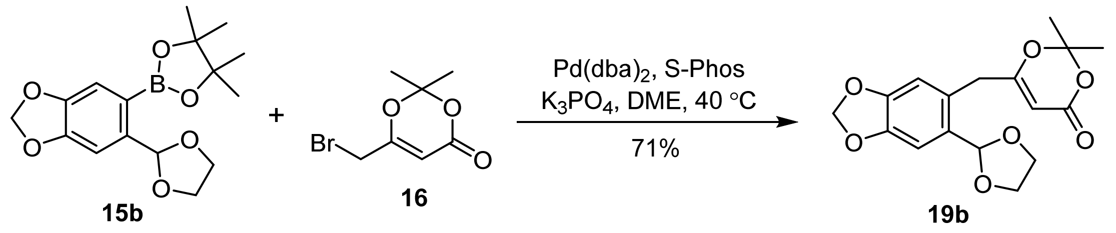

To a solution of pinacolyl borate **15b** (64.2 mg, 0.20 mmol) and alkyl bromide **16** (57.0 mg, 0.26 mmol) in DME (3 mL) was added Pd(dba)$_2$ (12.0 mg, 0.02 mmol), S-Phos (16.1 mg, 0.04 mmol) and K_3PO_4 (115.2 mg, 0.5 mmol) at room temperature under Ar. After being stirred at 40 ºC for 7 h, the resulting mixture was filtered through a short column of silica gel and washed with ethyl acetate (ca. 15 mL). The organic layer was concentrated under reduced pressure. The residue was purified by column chromatography on silica gel (petroleum ether/EtOAc = 4:1) to afford dioxinone **19b** (50.1 mg, 71%) as a pale-yellow syrup.

^{1}**H NMR (400 MHz, CDCl$_3$) δ ppm** 7.06 (s, 1H), 6.64 (s, 1H), 5.96 (s, 2H), 5.81 (s, 1H), 5.08 (s, 1H), 4.23 – 4.03 (m, 2H), 4.03 – 3.92 (m, 2H), 3.57 (s, 2H), 1.63 (s, 6H).

^{13}**C NMR (100 MHz, CDCl$_3$) δ ppm** 170.5, 161.4, 148.3, 147.2, 130.1, 126.4, 110.8, 107.2, 106.7, 101.5, 101.4, 94.4, 65.3, 36.3, 25.0.

HRMS (ESI): Calcd for $C_{17}H_{18}O_7$ $[M+Na]^+$: 357.0945, found: 357.0952.

2.12 Aldehyde 14b

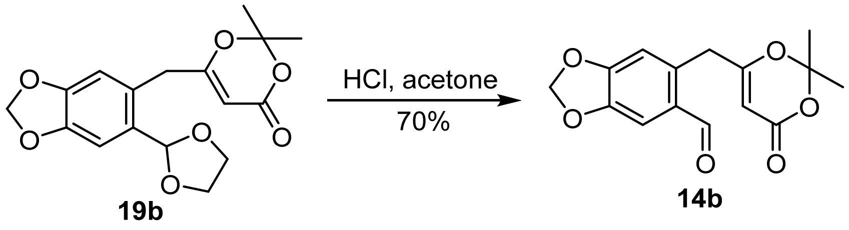

To a solution of dioxinone **19b** (545.0 mg, 1.63 mmol) in acetone (20 mL) was added HCl (2 N, 2.5 mL, 4.9 mmol) at room temperature. The reation mixture was allowed to stir at room temperature for 24 h. After TLC analysis, the reaction was quenched with saturated *aq*. $NaHCO_3$ (10 mL). The resulting mixture was then diluted with water (10 mL), and extracted with EtOAc (3 × 10 mL). The combined organic phases were washed with brine (20 mL), dried over anhydrous Na_2SO_4, filtered, and concentrated under reduced pressure. The residue was purified by column chromatography on silica gel (petroleum ether/EtOAc = 4:1) to give aldehyde **14b** (307.6 mg, 65%) as a white solid.

^{1}H NMR (400 MHz, $CDCl_3$) δ ppm 9.97 (s, 1H), 7.30 (s, 1H), 6.76 (s, 1H), 6.10 (s, 2H), 5.02 (d, J = 0.9 Hz, 1H), 3.91 (s, 2H), 1.65 (s, 6H).

^{13}C NMR (100 MHz, $CDCl_3$) δ ppm 189.8, 170.0, 161.2, 152.4, 147.9, 132.4, 129.0, 112.0, 111.9, 107.0, 102.5, 94.1, 36.5, 25.1.

HRMS (ESI): Calcd for $C_{15}H_{14}O_6$ $[M+Na]^+$: 313.0683, found: 313.0688.

2.13 Benzhydrol 12b

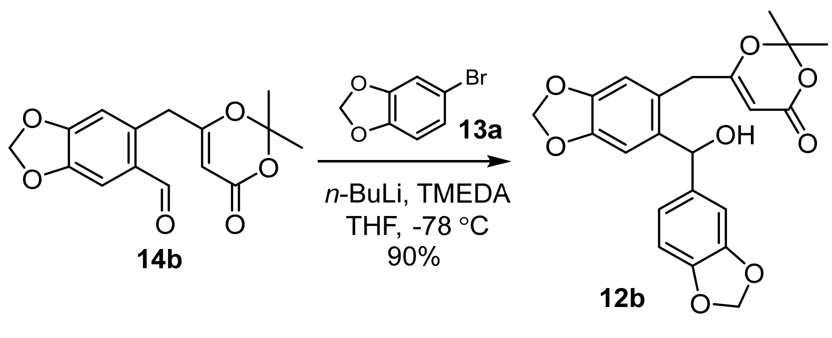

To a solution of aryl bromide **13a** (1.39 g, 6.90 mmol) and TMEDA (800.1 mg, 6.90 mmol) in dry THF (30 mL) was added *n*-BuLi (2.5 M, 2.8 mL 7.0 mmol) at -78 °C under Ar. The mixture stirred at -78 °C for 30 min. A solution of aldehyde **14b** (400.1 mg, 1.38 mmol) in THF (10 mL) was then added dropwise and the mixture allowed to stir for 3 h. The mixture was quenched with saturated *aq.* NH_4Cl (5 mL). The resulting mixture was then diluted with water (10 mL), and extracted with EtOAc (3 × 20 mL). The combined organic phases were washed with brine (30 mL), dried over anhydrous Na_2SO_4, filtered, and concentrated under reduced pressure. The residue was purified by column chromatography on silica gel (petroleum ether/EtOAc = 4:1) to give benzhydrol **12b** (511.4 mg, 90%) as a pale-yellow oil.

^{1}H NMR (400 MHz, $CDCl_3$) δ ppm 6.98 (s, 1H), 6.73 (s, 2H), 6.72 (s, 1H), 5.96 (s, 1H), 5.95 (s, 1H), 5.91 (s, 2H), 5.77 (s, 1H), 4.93 (s, 1H), 3.42 (s, 2H), 2.57 (brs, 1H), 1.60 (s, 6H).

^{13}C NMR (100 MHz, $CDCl_3$) δ ppm 170.4, 161.3, 148.0, 147.4, 147.2, 136.8, 136.0, 124.8, 120.4, 110.7, 108.3, 108.0, 107.5, 106.7, 101.5, 101.3, 94.2, 72.8, 36.4, 25.1, 24.9.

HRMS (ESI): Calcd for $C_{22}H_{20}O_8$ $[M+Na]^+$: 435.1050, found: 435.1047.

2.14 Dihydronaphthalene 11b

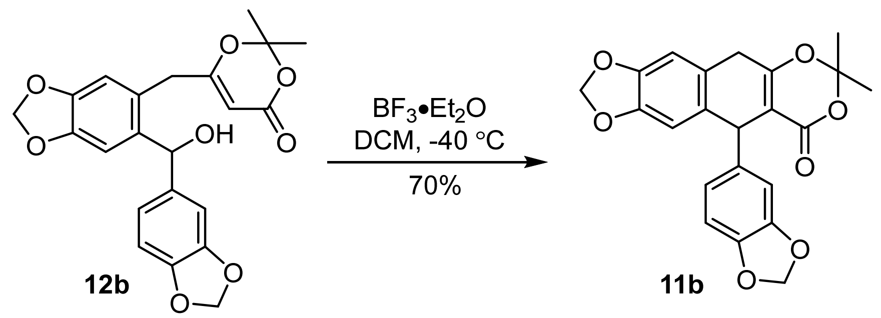

The benzhydrol **12b** (700.0 mg, 1.70 mmol) was dissolved in dry DCM (25 mL), and $BF_3•Et_2O$ (0.53 mL, 4.2 mmol) was added at –40 °C. After being stirred at –40 °C for 4 h, the reaction was quenched with saturated *aq.* $NaHCO_3$ (10 mL) and diluted with

water (20 mL). The aqueous layer was then extracted with DCM (3 × 20 mL). The combined organic phases were washed with brine (30 mL) and dried over anhydrous Na_2SO_4. After removal of the solvents under reduced pressure, the residue was purified by column chromatography on silica gel (petroleum ether/EtOAc = 5:1) to yield dihydronaphthalene **11b** (469.3 mg, 70%) as a white solid.

^{1}H NMR (400 MHz, $CDCl_3$) δ ppm 6.74 (s, 0.32H), 6.72 (s, 0.78H), 6.69 (s, 0.78H), 6.67 (s, 0.32H), 6.61 (s, 1H), 6.59 (s, 1H), 6.58 (s, 1H), 5.91 (s, 1H), 5.88 (s, 1H), 5.87 (s, 2H), 4.96 (s, 1H), 3.73 (d, J = 3.5 Hz, 0.4H), 3.68 (d, J = 3.4 Hz, 0.62H), 3.52 (d, J = 1.9 Hz, 0.64H), 3.46 (d, J = 1.9 Hz, 0.38H), 1.70 (s, 3H), 1.60 (s, 3H).

^{13}C NMR (100 MHz, $CDCl_3$) δ ppm 162.3, 160.5, 147.9, 147.18, 146.8, 146.3, 139.0, 130.7, 122.7, 121.1, 108.9, 108.4, 108.1, 107.4, 106.2, 105.4, 101.2, 101.0, 43.7, 32.4, 27.0, 23.9.

HRMS (ESI): Calcd for $C_{22}H_{18}O_7$ $[M+Na]^+$: 417.0945, found: 417.0945.

2.15 Triflate 10b

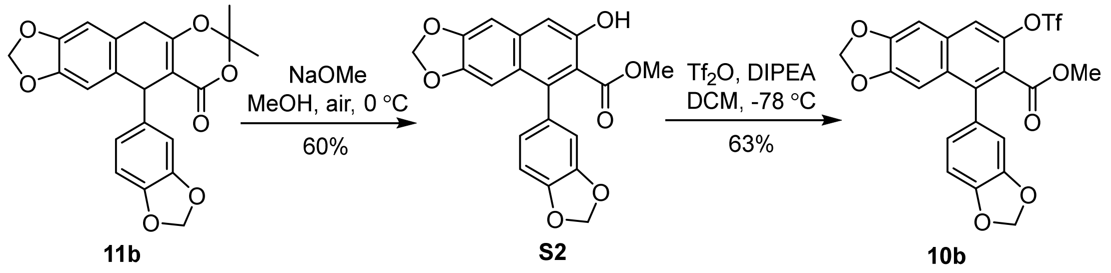

Sodium (443.6 mg, 19.3 mmol) was added portionwise to methanol (10 mL) at 0 °C. After addition, the reaction flask was removed from the ice bath and warmed up to room temperature. After being stirred at room temperature for 30 min, compound **11b** (380.0 mg, 0.96 mmol) was introduced to the flask in one portion, and the reaction mixture was then stirred under air for 12 h. After TLC analysis, saturated *aq.* NH_4Cl (8 mL) was added to the reaction mixture at 0 °C, and the resulting mixture was then extracted with EtOAc (3 × 15 mL). The combined organic phases were washed with brine (30 mL), dried over anhydrous Na_2SO_4, filtered, and concentrated under reduced

pressure. The residue was purified by column chromatography on silica gel (petroleum ether/EtOAc = 6:1) to give methyl ester **S2** (288.2 mg, 60%) as a pale-yellow solid. To a solution of methyl ester **S2** (300.0 mg, 0.82 mmol) in anhydrous DCM (15 mL) was added DIPEA (0.60 mL, 3.26 mmol) at –78 °C. After being stirred at –78 °C for 10 min, Tf_2O (0.27 mL, 1.63 mmol) was introduced. The reaction mixture was then stirred at –78 °C for 3 h before being quenched with saturated *aq.* $NaHCO_3$ (10 mL). The aqueous layer was extracted with DCM (3 × 10 mL), and the combined organic phases were dried over anhydrous Na_2SO_4. After removal of the solvents under reduced pressure, the residue was purified by column chromatography on silica gel (petroleum ether/EtOAc = 6:1) to give triflate **10b** (256.0 mg, 63%) which was used directly in the next reaction.

HRMS (ESI): Calcd for $C_{20}H_{14}O_7$ $[M+Na]^+$: 389.0632, found: 389.0634.

2.16 Taiwanin C (4)

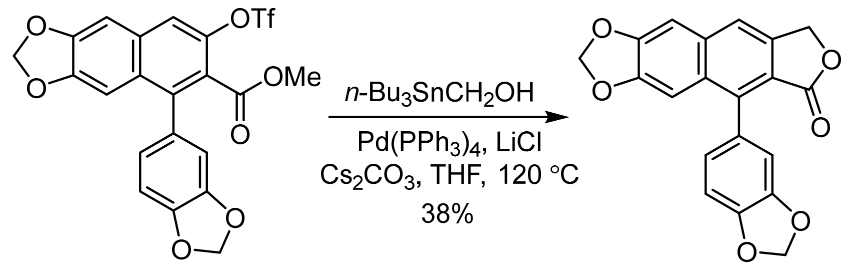

A flame-dried 10 mL sealed tube was sequentially charged with **10b** (50.0 mg, 0.1 mmol), LiCl (17.1 mg, 0.4 mmol), Cs_2CO_3 (65.2 mg, 0.2 mmol), tributylstannyl methanol (38.6 mg, 0.12 mmol) and Pd(PPh3)4 (11.6 mg, 0.01 mmol). And anhydrous THF (5 mL) was then added into the flask. The tube was sealed with a terflon screw top and the mixture was heated at 120 °C under Ar for 12 h. The saturated *aq.* $NaHCO_3$ (5 mL) was then added to the reaction mixture at rt. The resulting mixture was then extracted with EtOAc (3 × 7 mL). The combined organic phases were washed with brine (10 mL), dried over Na_2SO_4, filtered, and concentrated under reduced pressure.

The residue was purified by flash chromatography on silica gel (petroleum ether/EtOAc = 5:1) to afford Taiwanin C (13.3 mg, 38%).

^{1}H NMR (400 MHz, DMSO) δ ppm 7.94 (s, 1H), 7.51 (s, 1H), 7.04 (d, *J* = 8.0 Hz, 1H), 6.89 (s, 1H), 6.87 (d, *J* = 1.5 Hz, 1H), 6.74 (dd, *J* = 7.9, 1.6 Hz, 1H), 6.18 (s, 1H), 6.17 (s, 1H), 6.17 (s, 1H), 6.13 (s, 2H), 5.43 (s, 2H).

^{13}C NMR (100 MHz, DMSO) δ ppm 169.3, 149.7, 148.5, 147.1, 147.1, 140.3, 138.8, 134.4, 129.5, 128.4, 123.4, 119.7, 118.5, 110.5, 108.1, 103.8, 102.2, 102.2, 101.2, 68.1.

HRMS (ESI): Calcd for $C_{20}H_{12}O_6$ $[M+Na]^+$: 371.0526, found: 371.0526.

2.17 Alcohol 16

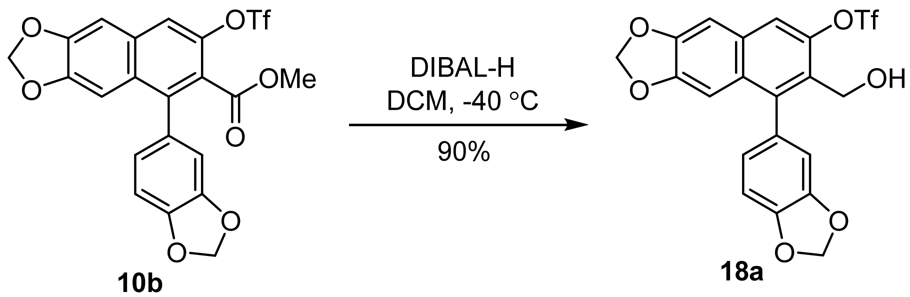

10b

18a

To a solution of compound **10b** (55.0 mg, 0.11 mmol) in dry DCM (10 mL) was added DIBAL-H (0.55 mL, 0.55 mmol) at -40 °C. The reaction mixture was stirred at room temperature under Ar for 4 h before being quenched with saturated *aq.* Rochelle salt (4 mL) and diluted with water (5 mL). The mixture was extracted with DCM (3 × 8 mL). The combined organic phases were washed with brine (10 mL), dried over Na_2SO_4, filtered, and concentrated. The residue was purified by column chromatography on silica gel (petroleum ether/EtOAc = 6:1) to yield alcohol **18a** (34.7 mg, 63%).

^{1}H NMR (400 MHz, $CDCl_3$) δ ppm 7.61 (s, 1H), 7.12 (s, 1H), 6.94 (d, *J* = 7.8 Hz, 1H), 6.79 (d, *J* = 1.6 Hz, 1H), 6.78 (s, 1H), 6.76 (dd, *J* = 7.8, 1.6 Hz, 1H), 6.07 (d, *J* = 1.3 Hz, 1H), 6.05 (d, *J* = 1.2 Hz, 1H), 6.02 (s, 2H), 4.59 (s, 2H), 1.88 (brs, 1H).

^{13}C NMR (100 MHz, $CDCl_3$) δ ppm 149.1, 149.0, 148.0, 147.7, 145.4, 141.8, 130.7, 130.6, 130.0, 127.9, 123.6, 118.5, 110.8, 108.6, 103.9, 103.7, 101.8, 101.5, 57.9.

HRMS (ESI): Calcd for $C_{20}H_{13}F_3O_8S$ $[M+Na]^+$: 493.0175, found: 493.0182.

2.18 Justicidin E (7)

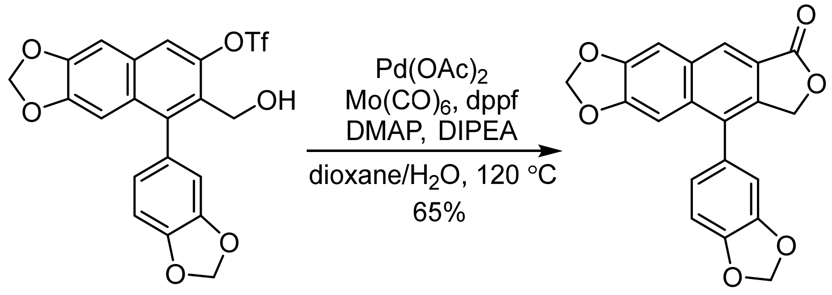

18a Justicidin E (**7**)

A flame-dried 10 mL sealed tube was sequentially charged with alcohol **18a** (47.0 mg, 0.1 mmol), $Mo(CO)_6$ (13.1 mg, 0.05 mmol), $Pd(OAc)_2$ (3.0 mg, 0.01 mmol), DMAP (24.0 mg, 0.2 mmol), DIPEA (47 μL, 0.25 mmol) and dppf (6.0 mg, 0.01 mmol). And dioxane/H_2O (V/V = 5:1, 5 mL) was then added into the flask. The tube was sealed with a terflon screw top and the mixture was heated at 120 °C under Ar for 5 h. The saturated *aq.* NH_4Cl (5 mL) was then added to the reaction mixture at rt. The resulting mixture was then extracted with EtOAc (3 × 8 mL). The combined organic phases were washed with brine (10 mL), dried over Na_2SO_4, filtered, and concentrated under reduced pressure. The residue was purified by flash chromatography on silica gel (petroleum ether/EtOAc = 8:1) to afford Justicidin E (22.1 mg, 65%).

^{1}H NMR (400 MHz, DMSO) δ ppm 8.33 (s, 1H), 7.61 (s, 1H), 7.08 (d, J = 7.9 Hz, 1H), 7.02 (d, J = 1.6 Hz, 1H), 6.99 (s, 1H), 6.89 (dd, J = 7.9, 1.7 Hz, 1H), 6.18 (s, 1H), 6.18 (s, 1H), 6.12 (s, 2H), 5.31 (d, J = 14.8 Hz, 1H), 5.25 (d, J = 14.8 Hz, 1H).

^{13}C NMR (100 MHz, DMSO) δ ppm 170.8, 150.4, 148.0, 147.8, 147.3, 138.6, 132.6, 132.1, 131.0, 129.1, 124.1, 123.0, 121.1, 109.7, 108.9, 105.2, 102.2, 101.4, 100.9, 69.2.

HRMS (ESI): Calcd for $C_{20}H_{12}O_6$ $[M+H]^+$: 349.0707, found: 349.0710.

3 Copies of ^{1}H and ^{13}C NMR Spectra

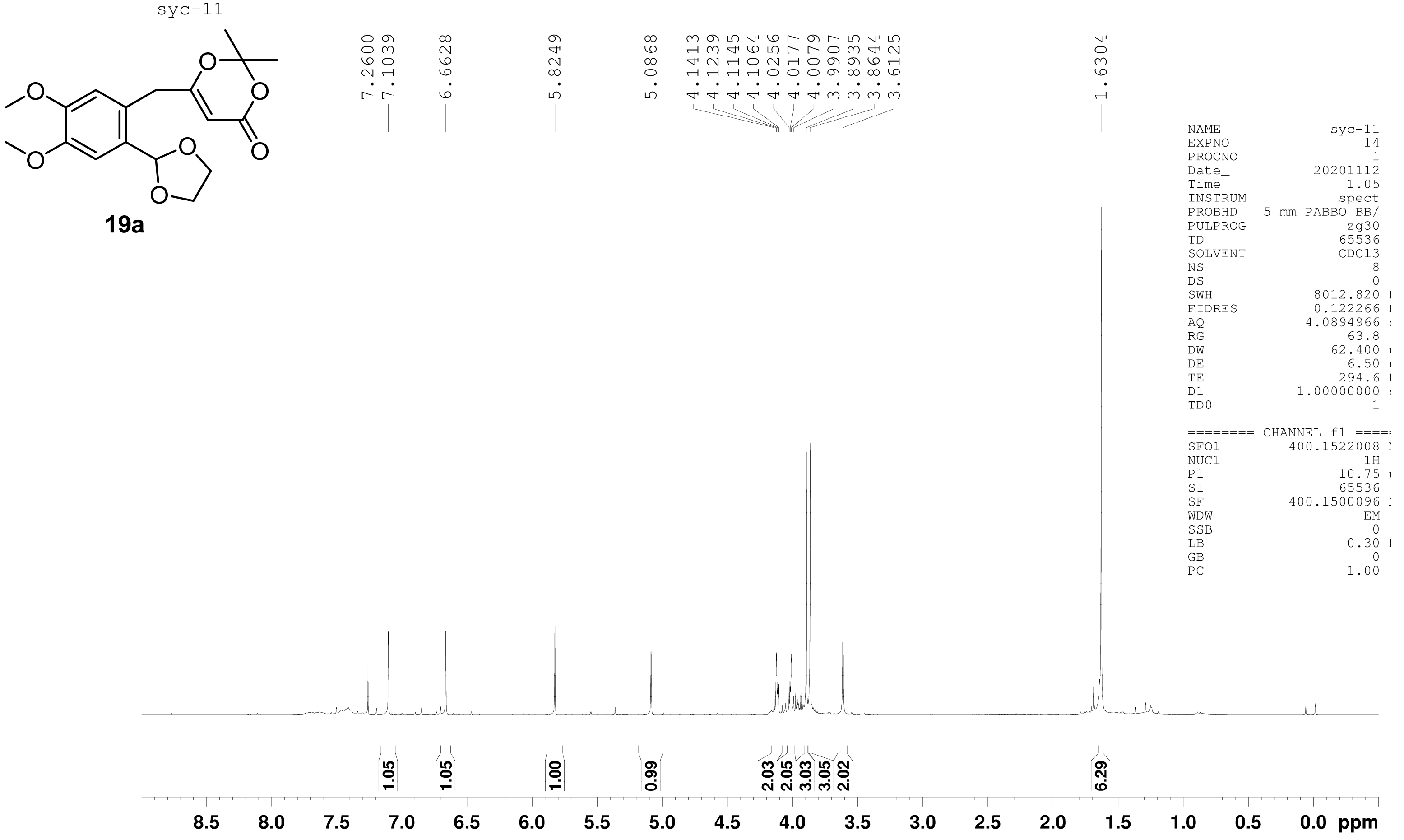

Figure S1 ^{1}H-NMR (400 MHz, $CDCl_3$) spectra of **19a**

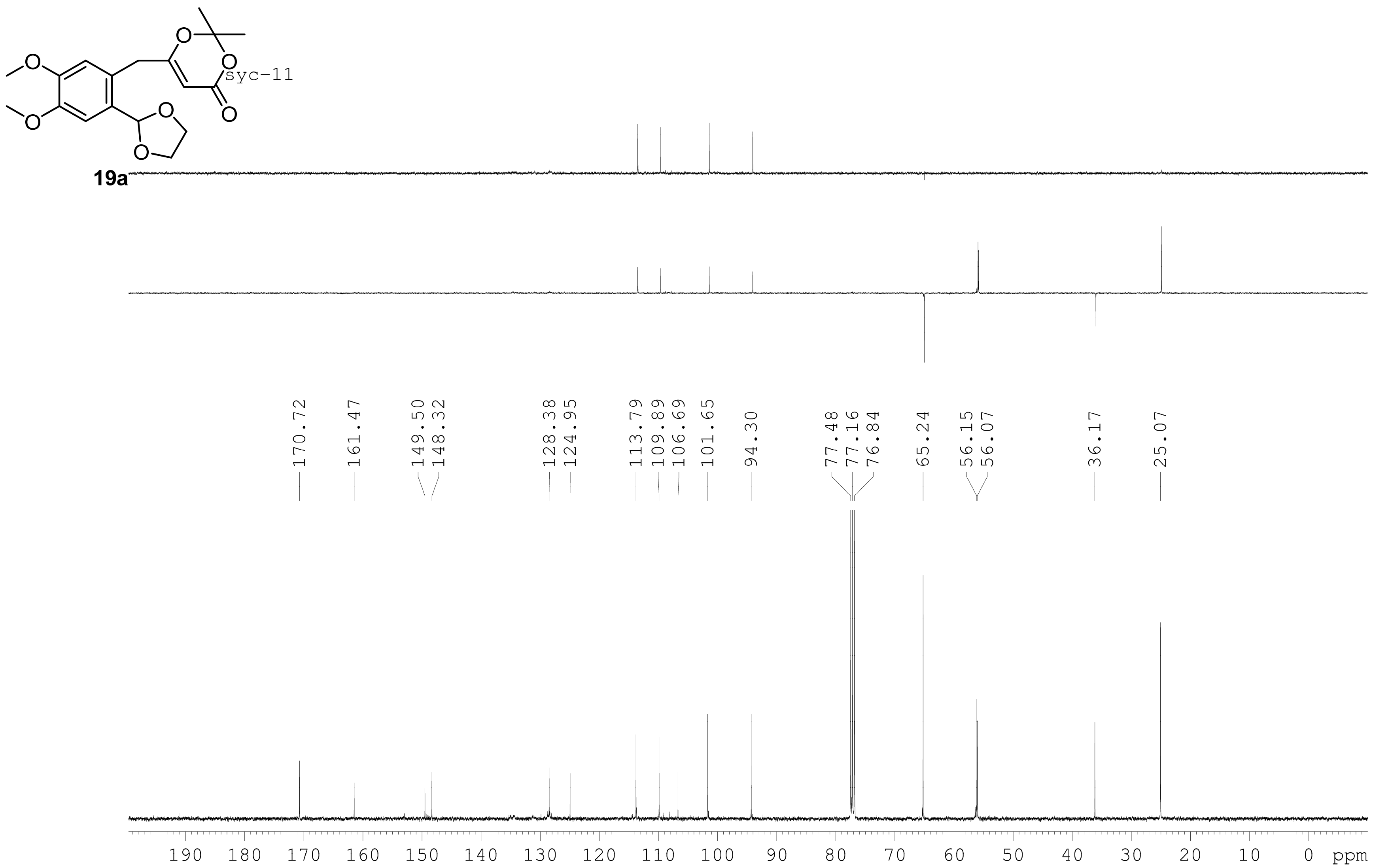

Figure S2 ^{13}C-NMR (100 MHz, $CDCl_3$) spectra of **19a**

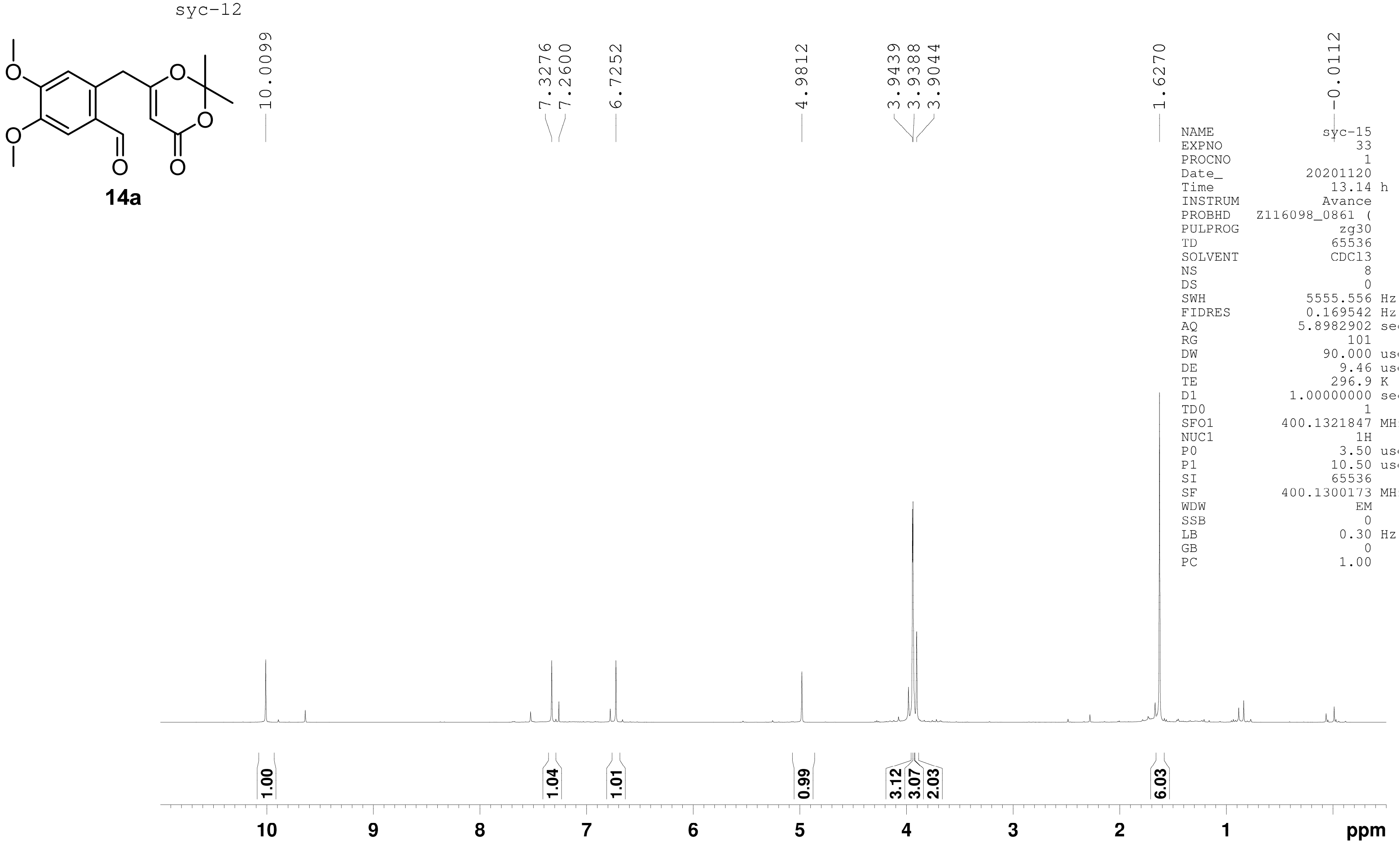

Figure S3 ^{1}H-NMR (400 MHz, $CDCl_3$) spectra of **14a**

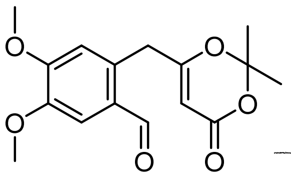

14a

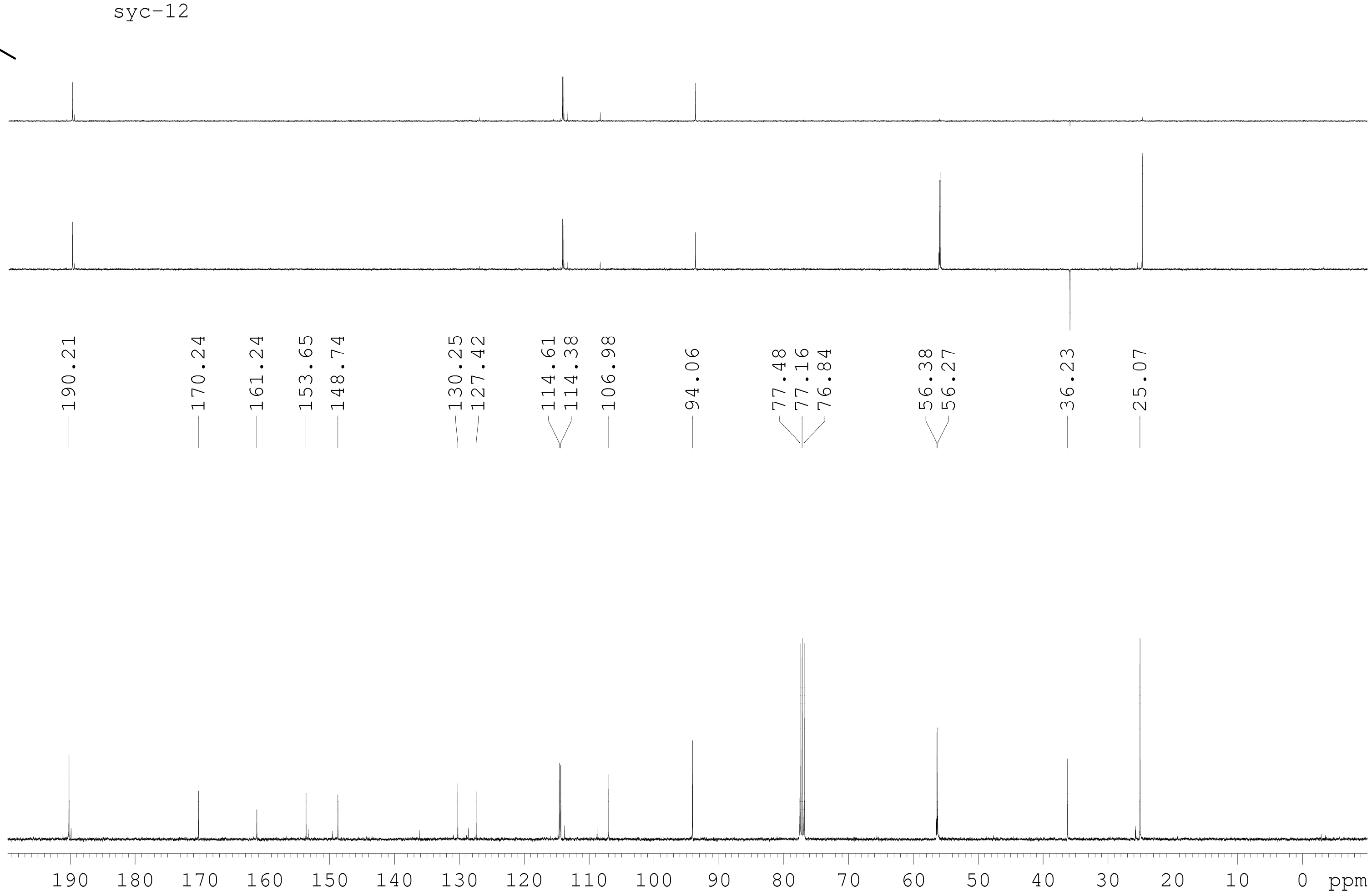

Figure S4 ^{13}C-NMR (100 MHz, $CDCl_3$) spectra of **14a**

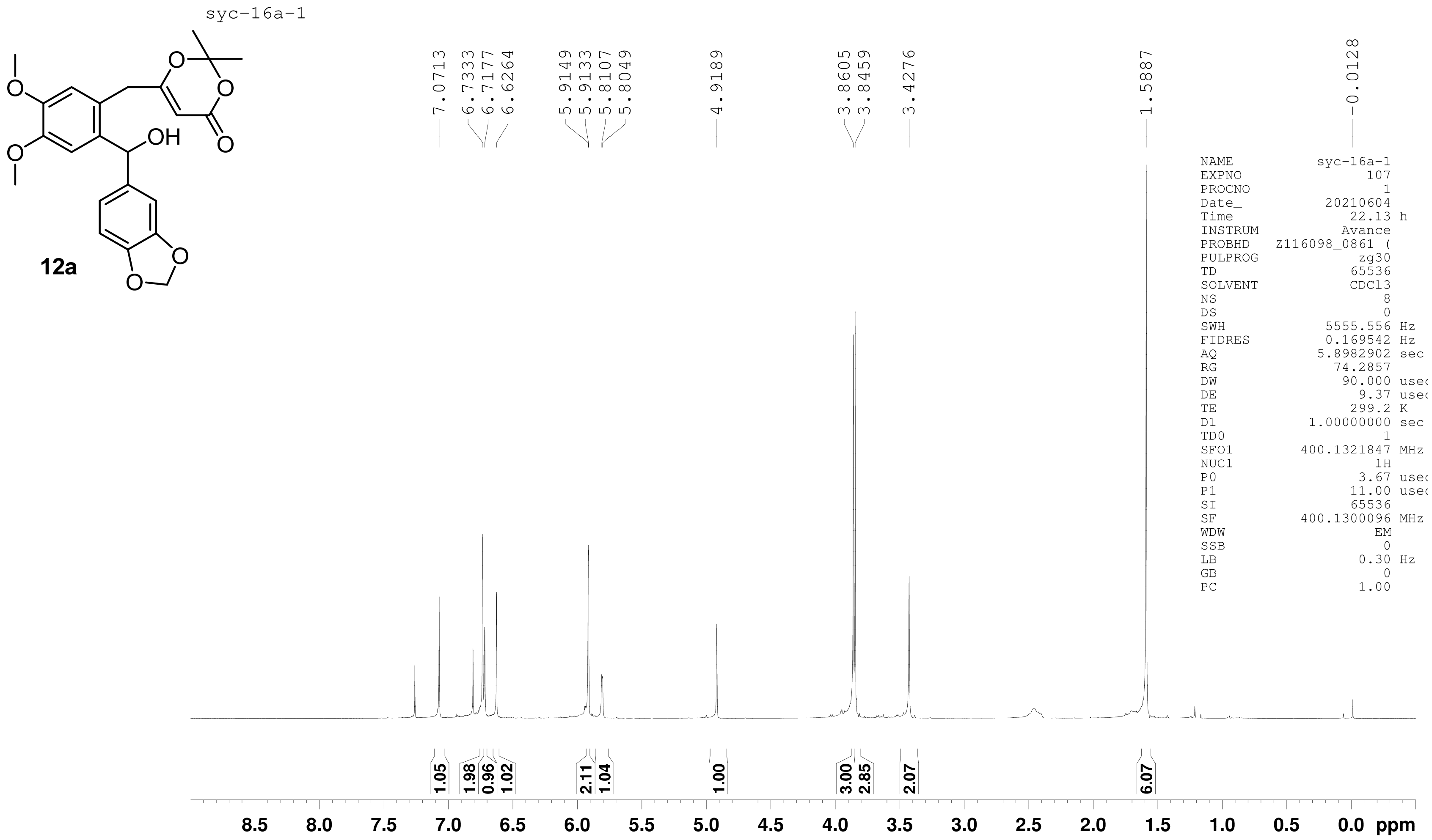

Figure S5 ^{1}H-NMR (400 MHz, $CDCl_3$) spectra of **12a**

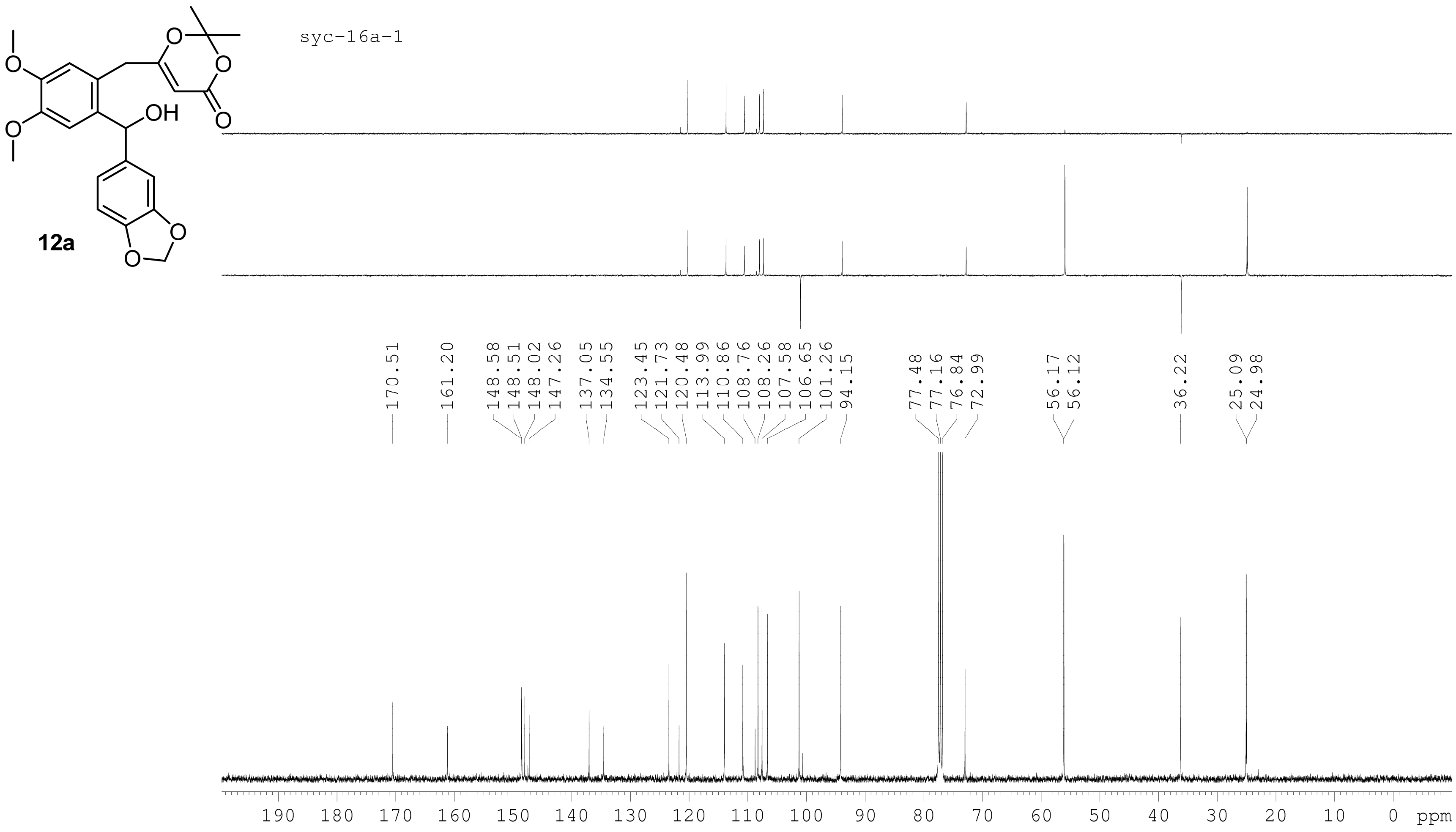

Figure S6 ^{13}C-NMR (100 MHz, $CDCl_3$) spectra of **12a**

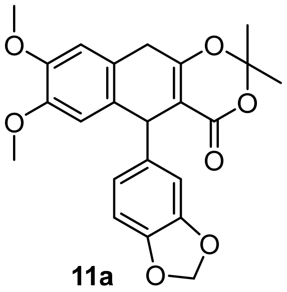

11a

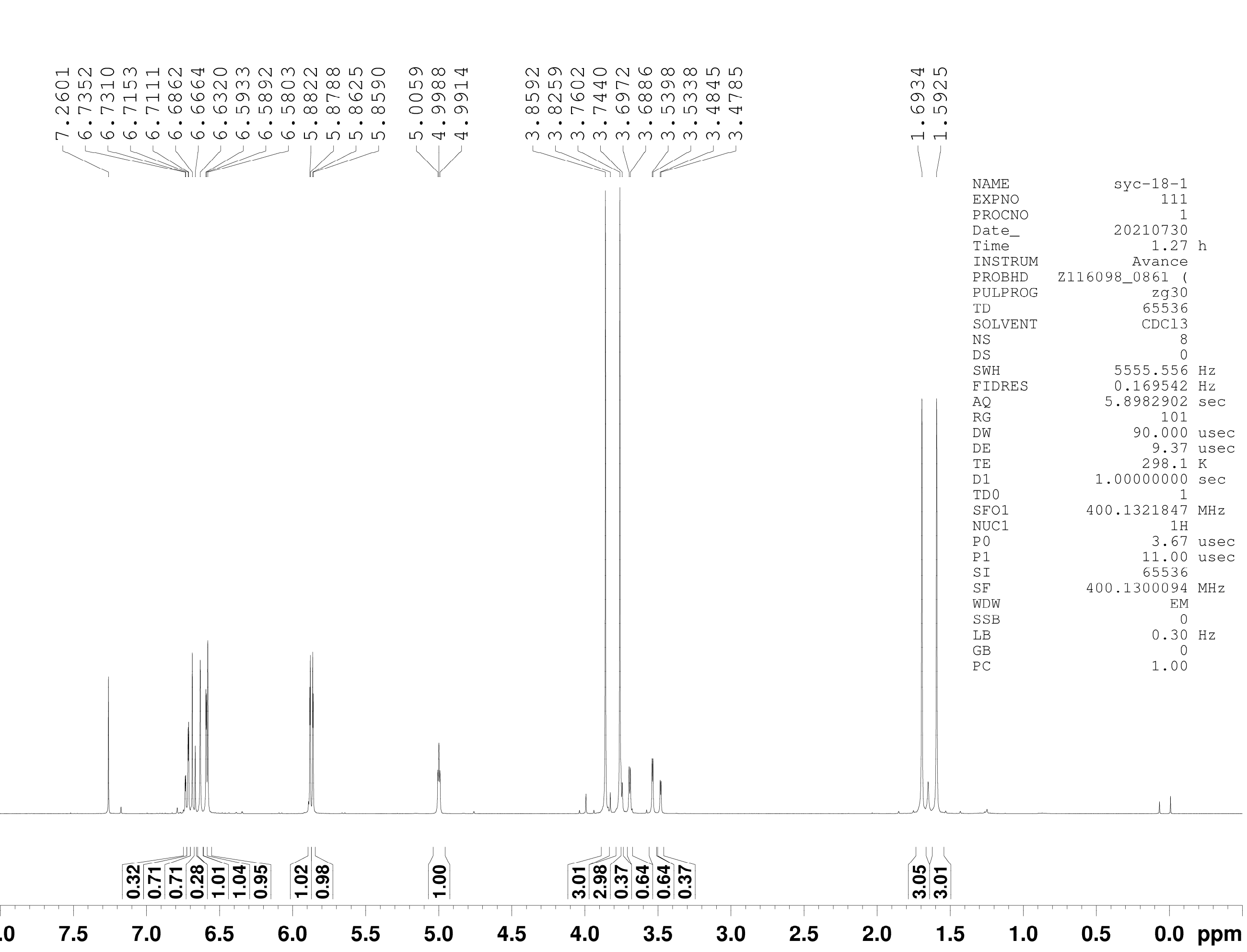

Figure S7 ^{1}H-NMR (400 MHz, $CDCl_3$) spectra of **11a**

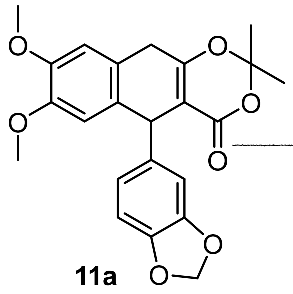

11a

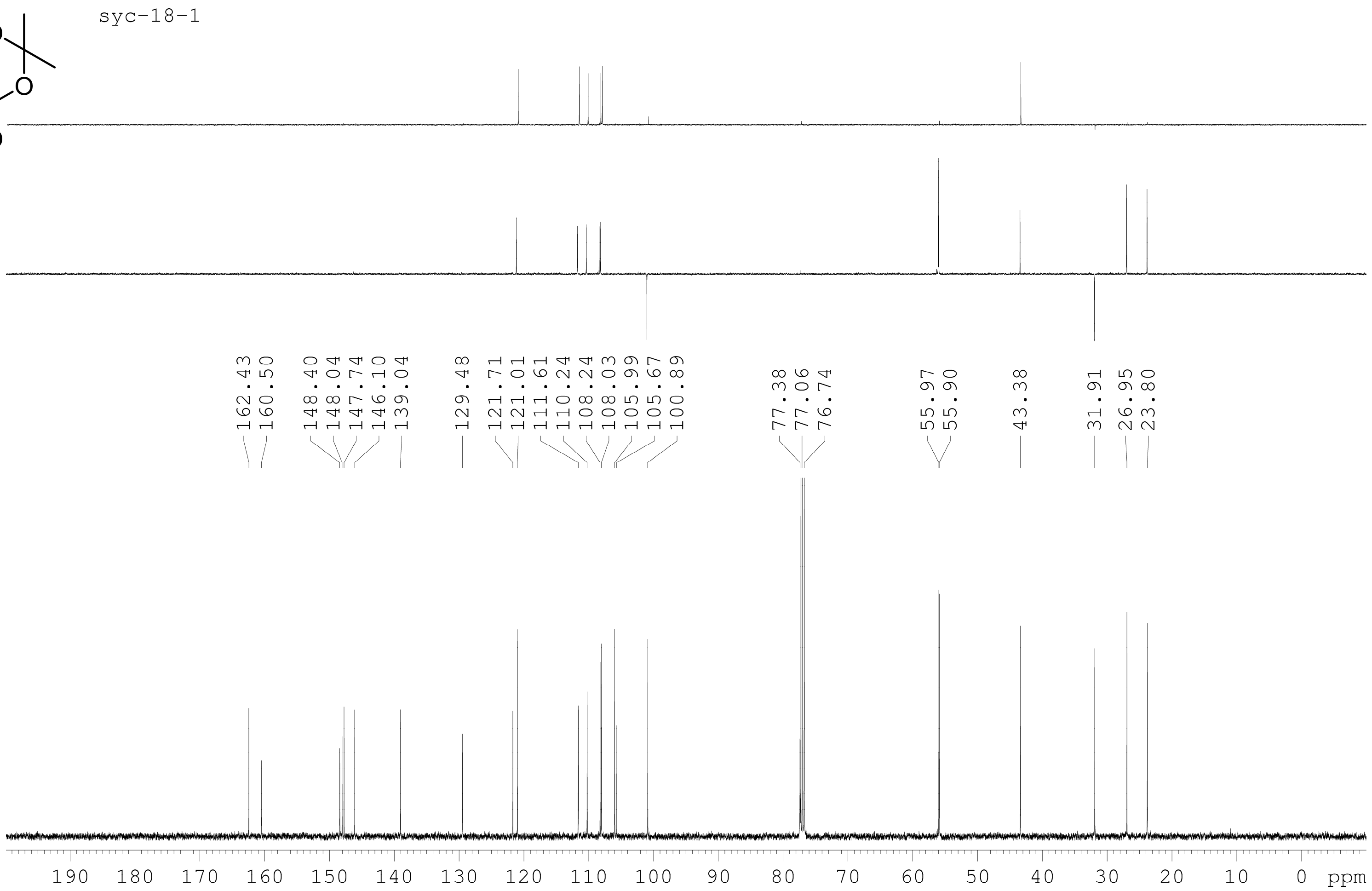

Figure S8 ^{13}C-NMR (100 MHz, $CDCl_3$) spectra of **11a**

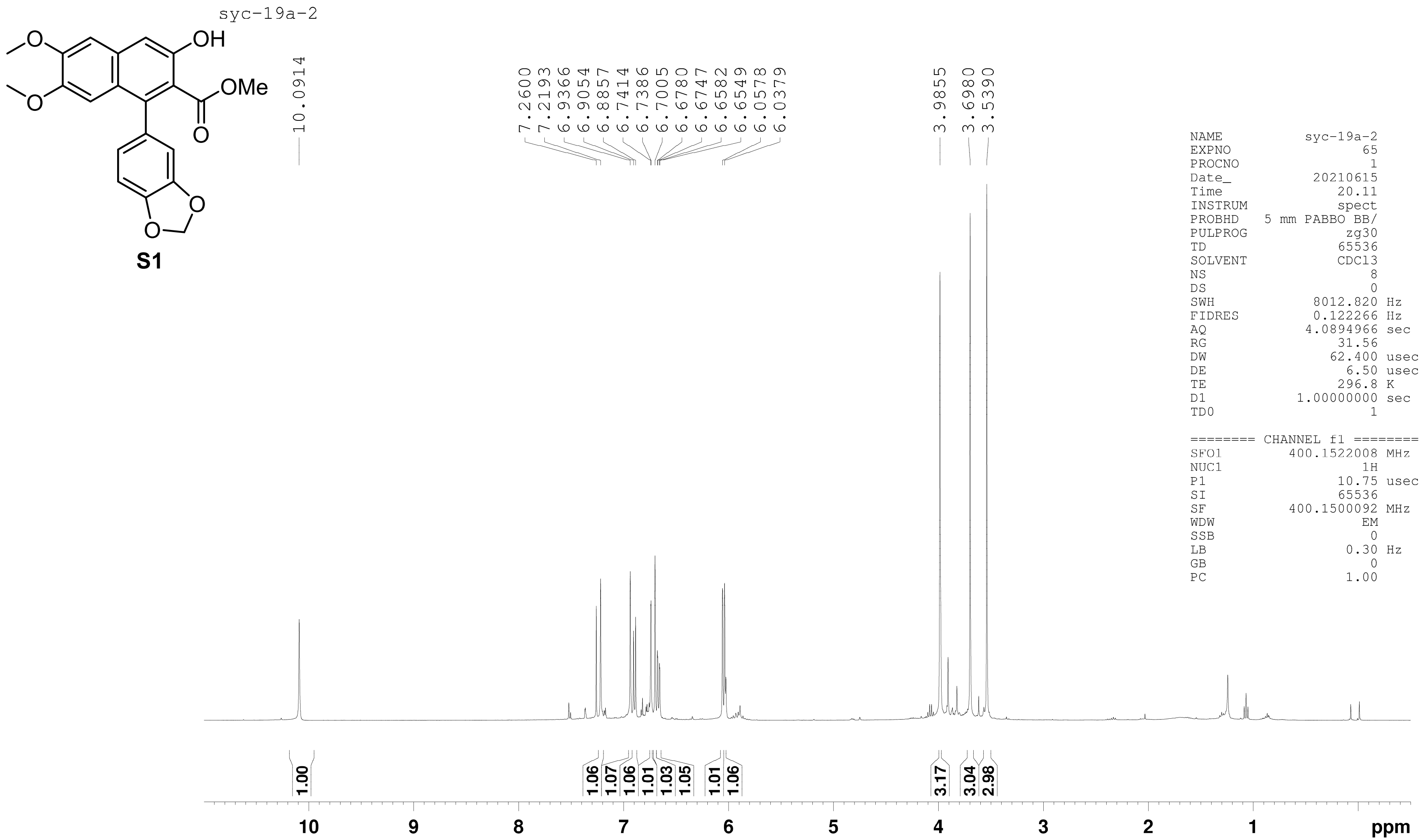

Figure S9 ^{1}H-NMR (400 MHz, $CDCl_3$) spectra of **S1**

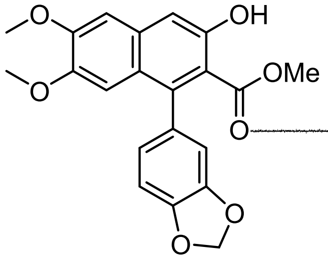

S1

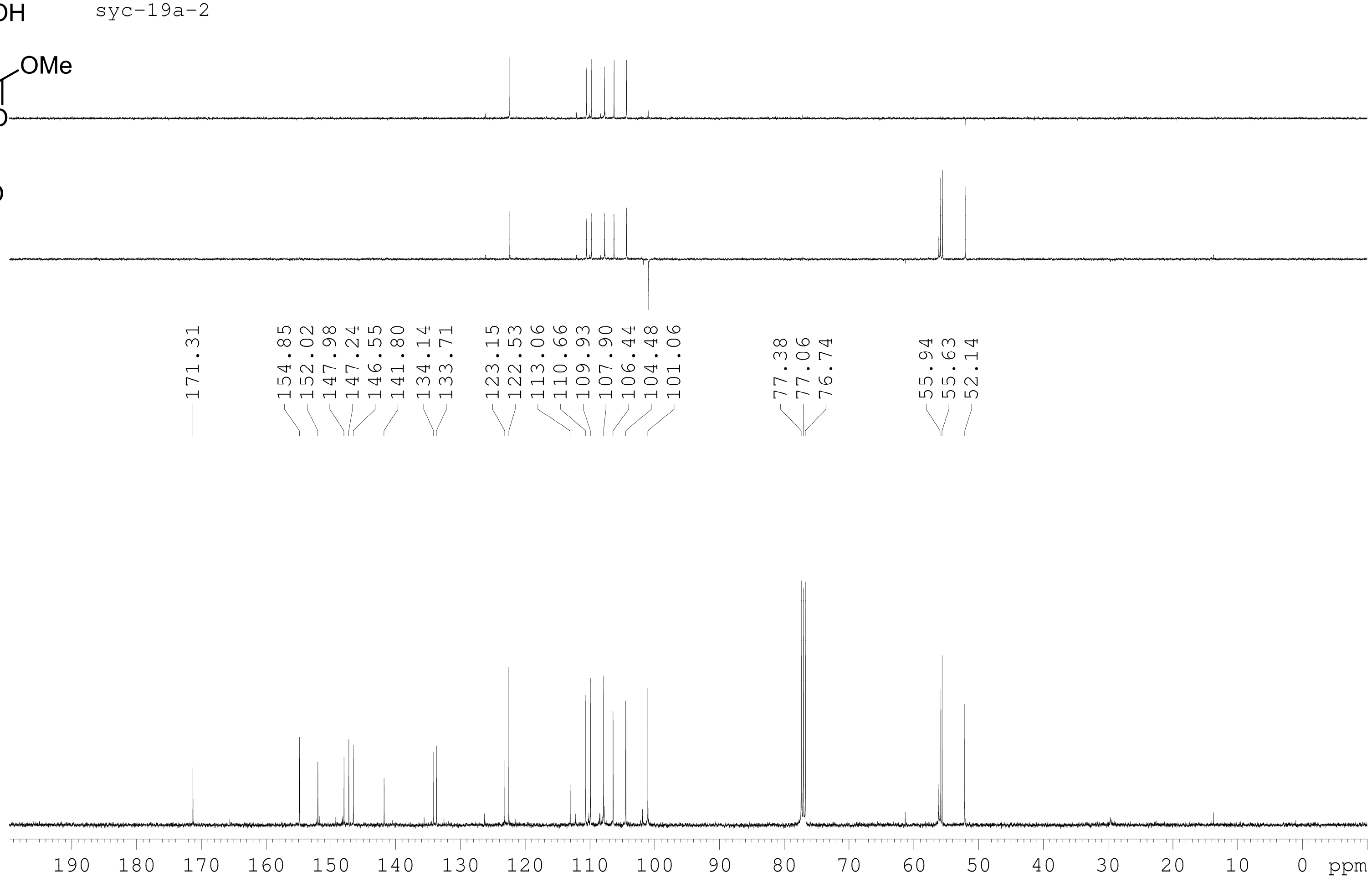

Figure S10 ^{13}C-NMR (100 MHz, $CDCl_3$) spectra of **S1**

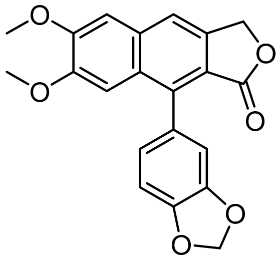

Justicidin B (**1**)

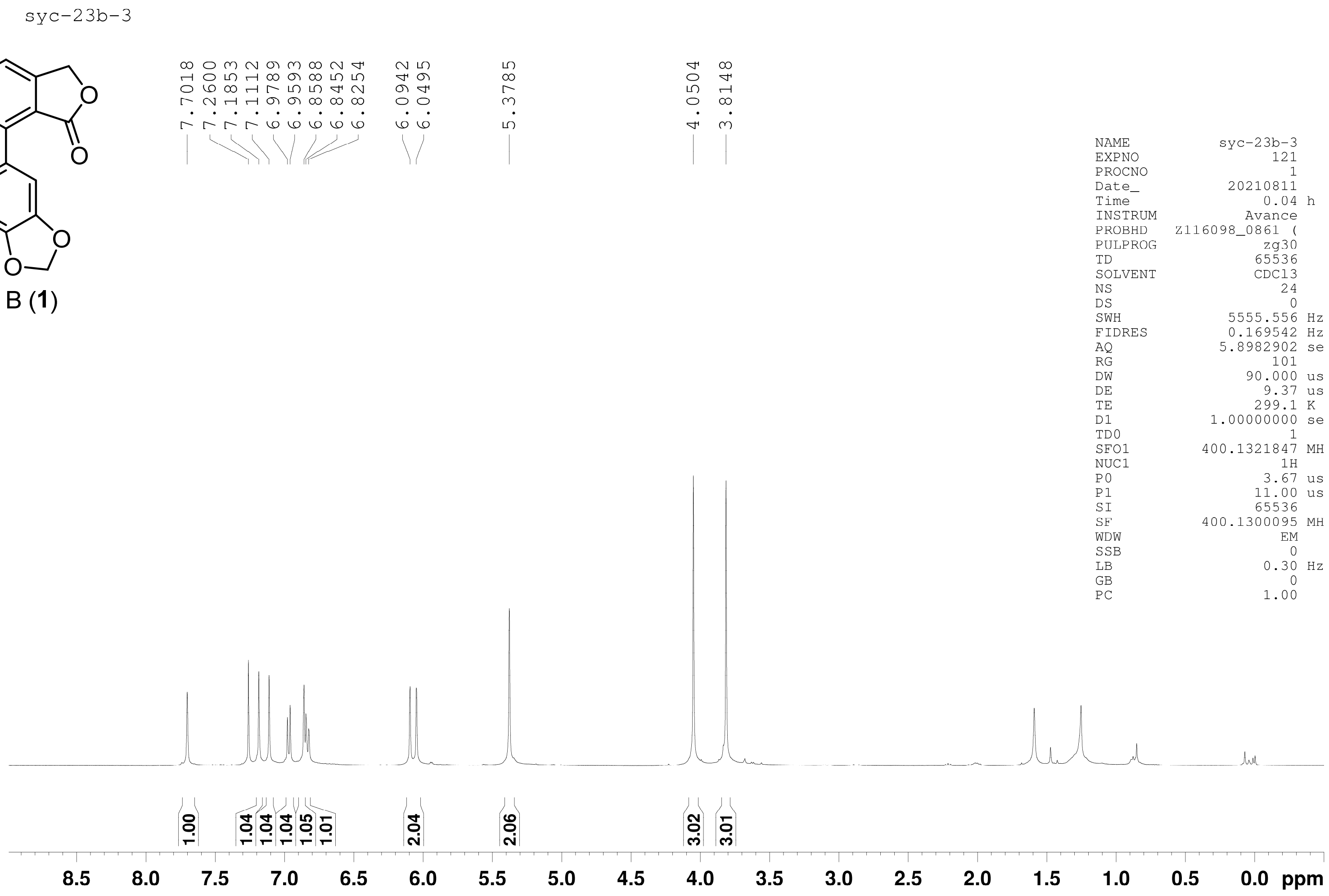

Figure S11 ^{1}H-NMR (400 MHz, $CDCl_3$) spectra of Justicidin B (**1**)

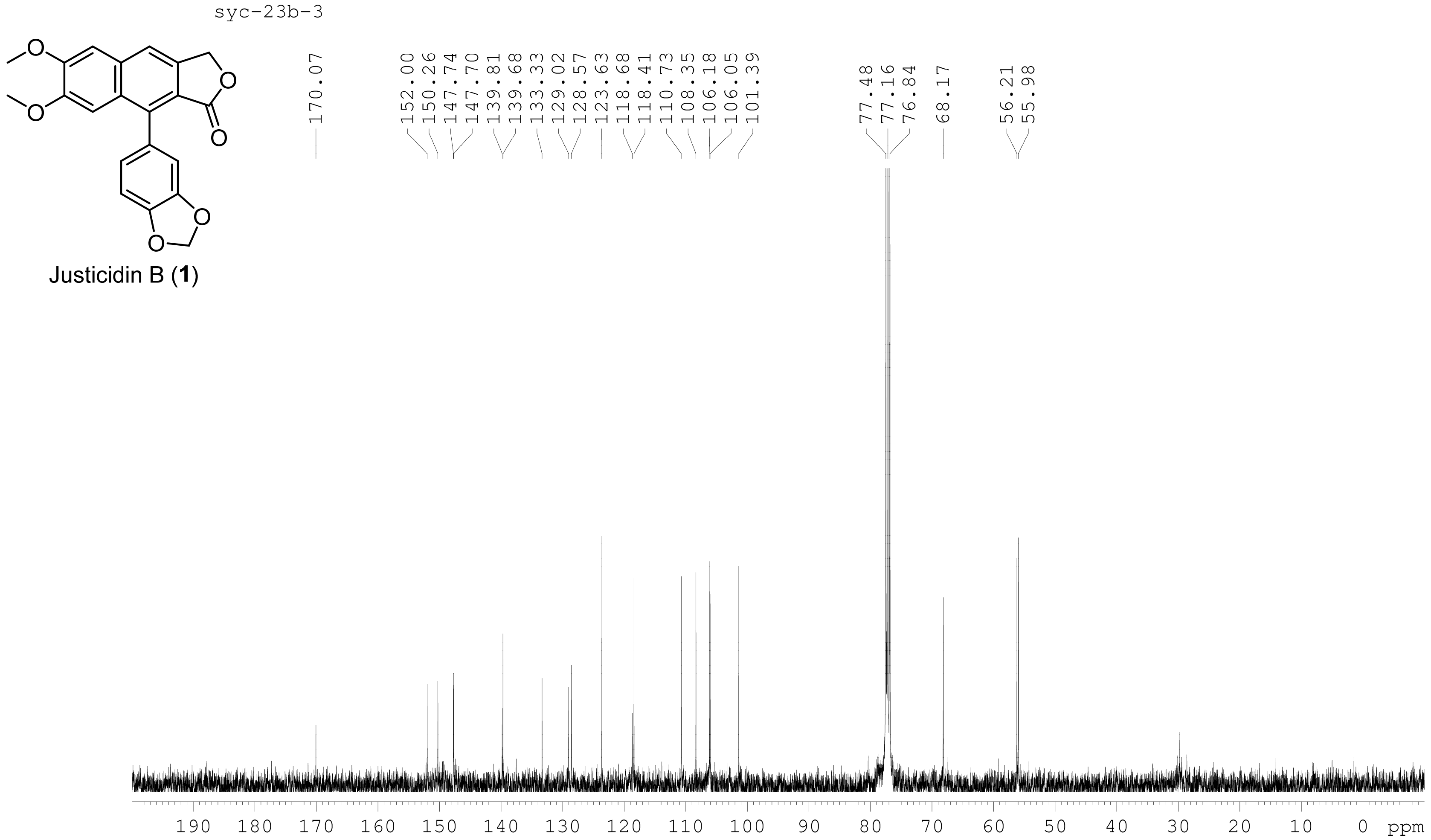

Figure S12 ^{13}C-NMR (100 MHz, $CDCl_3$) spectra of Justicidin B (**1**)

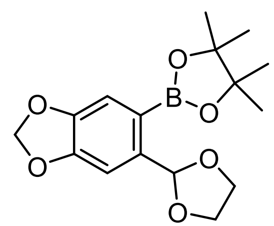

15b

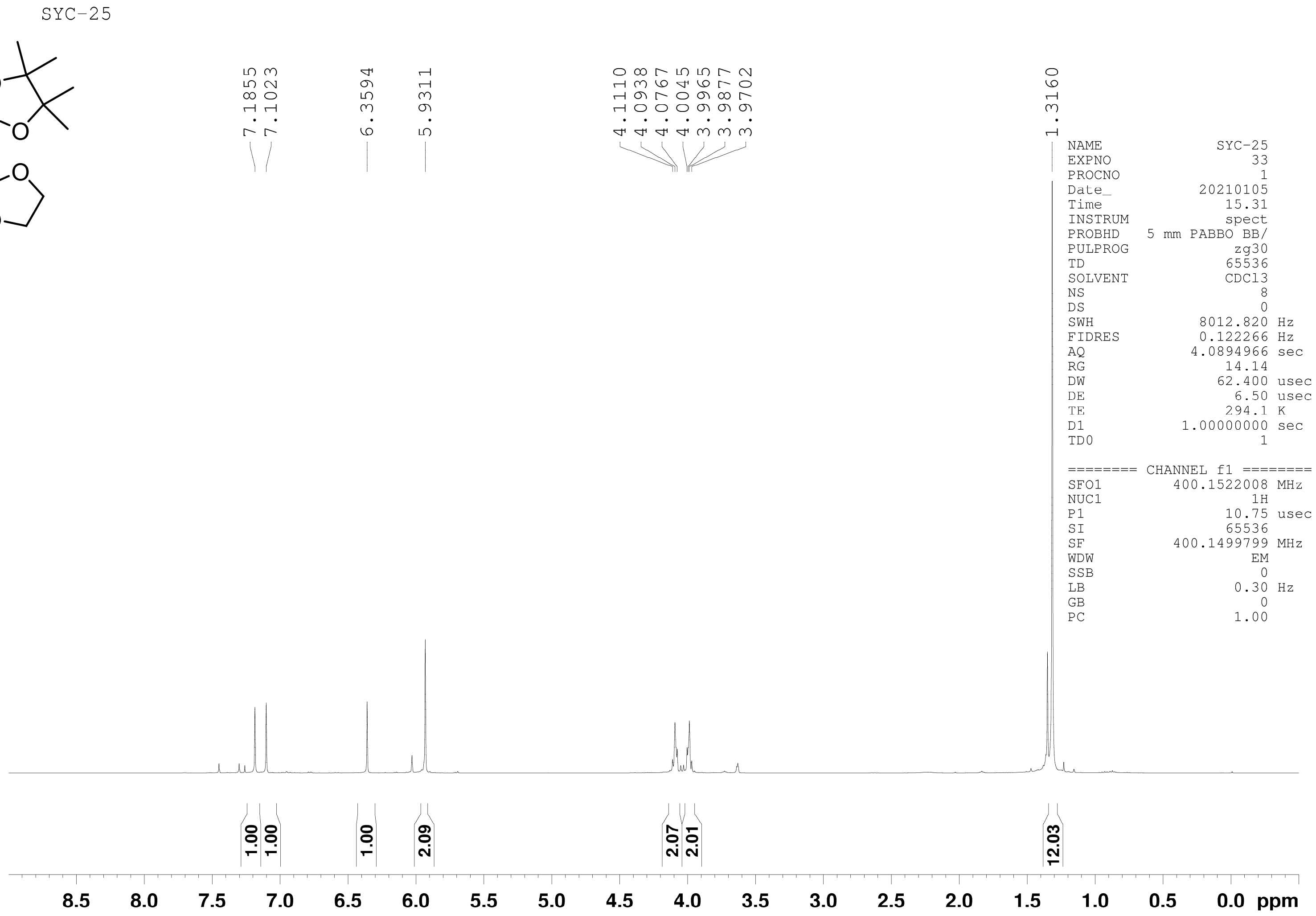

Figure S13 ^{1}H-NMR (400 MHz, $CDCl_3$) spectra of **15b**

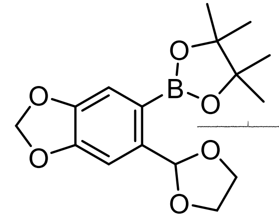

15b

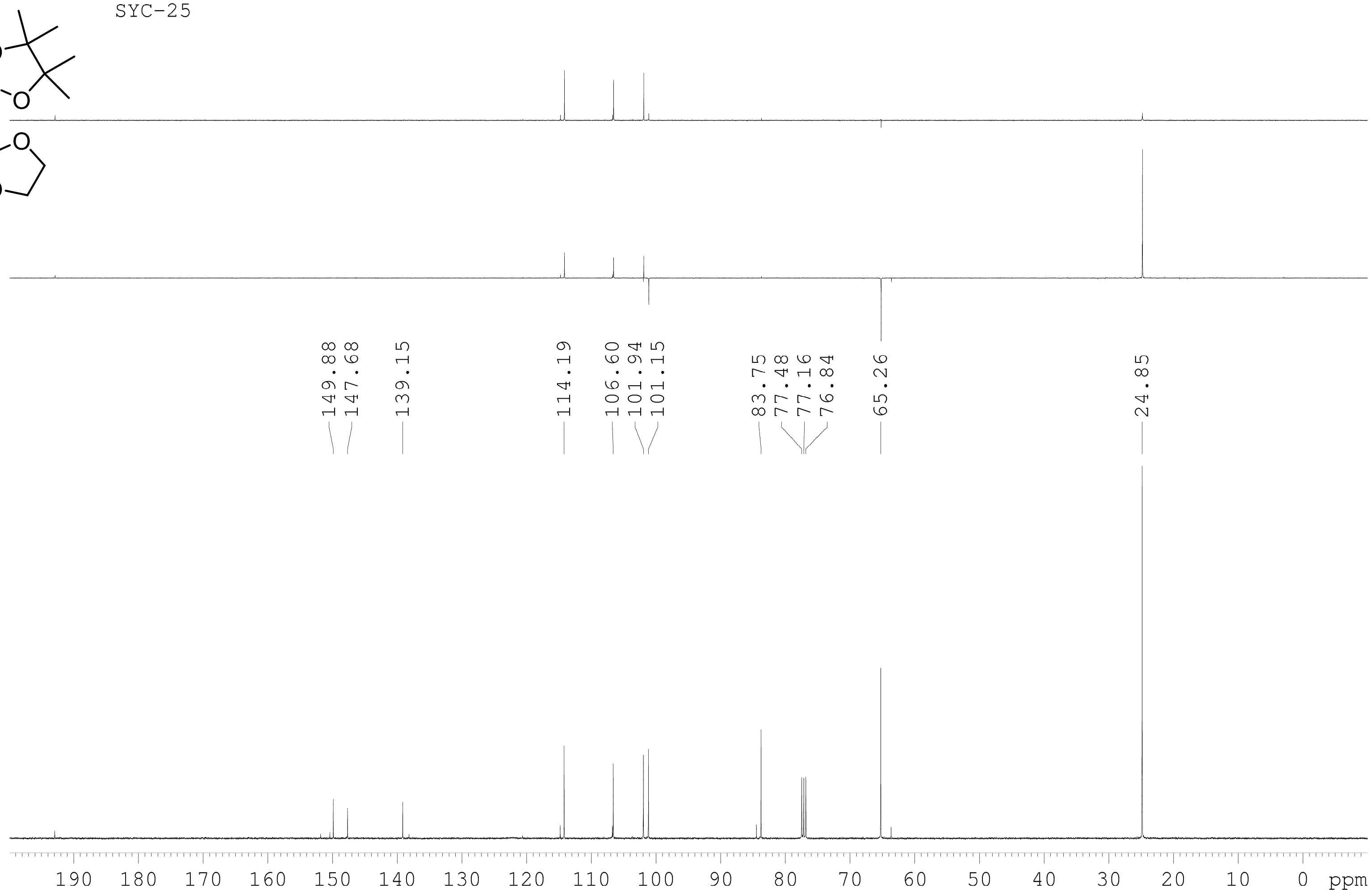

Figure S14 ^{13}C-NMR (100 MHz, $CDCl_3$) spectra of **15b**

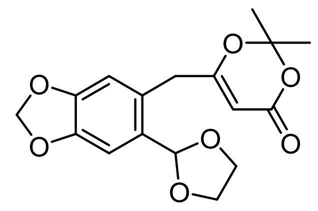

19b

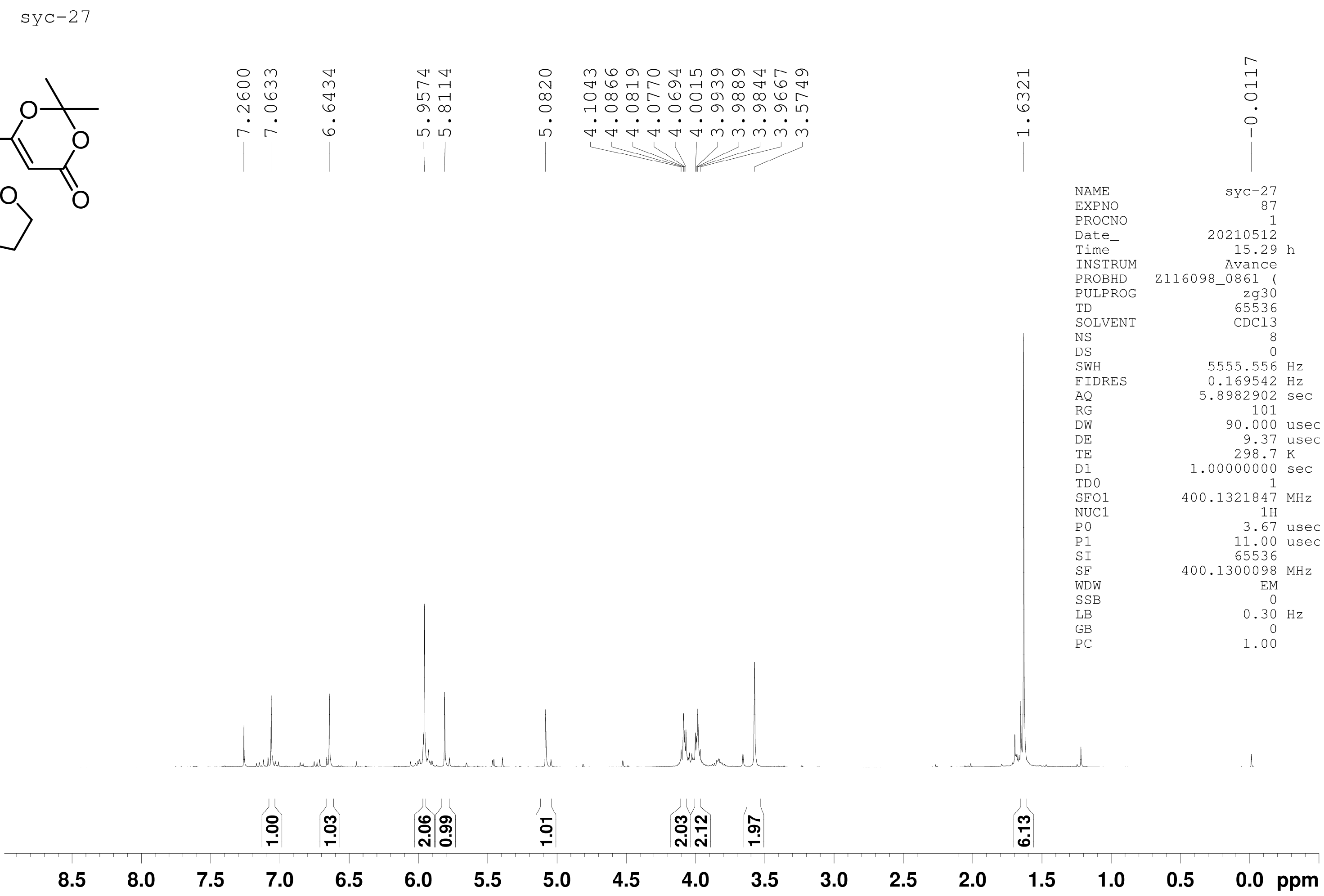

Figure S15 ^{1}H-NMR (400 MHz, $CDCl_3$) spectra of **19b**

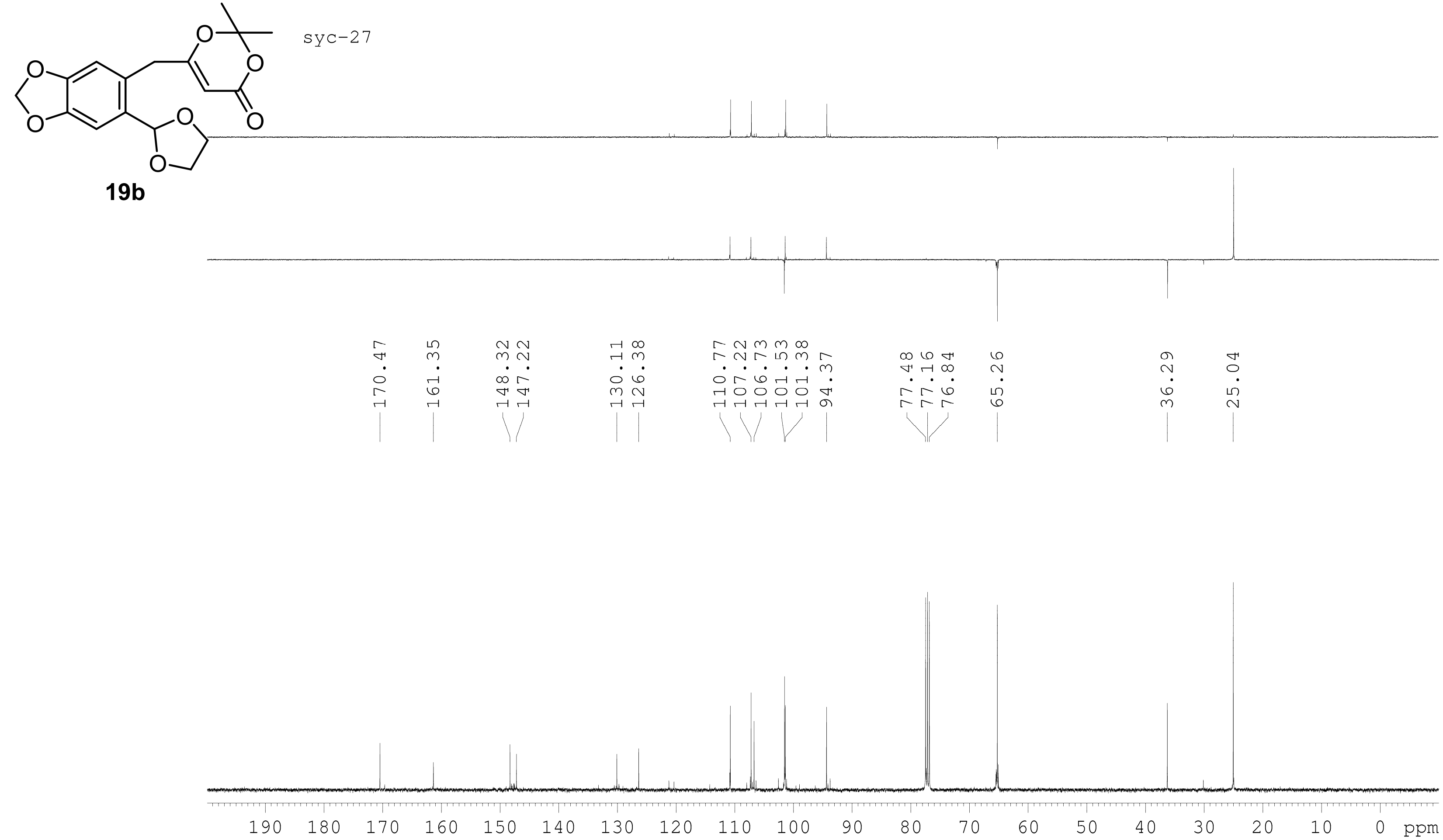

Figure S16 ^{13}C-NMR (100 MHz, $CDCl_3$) spectra of **19b**

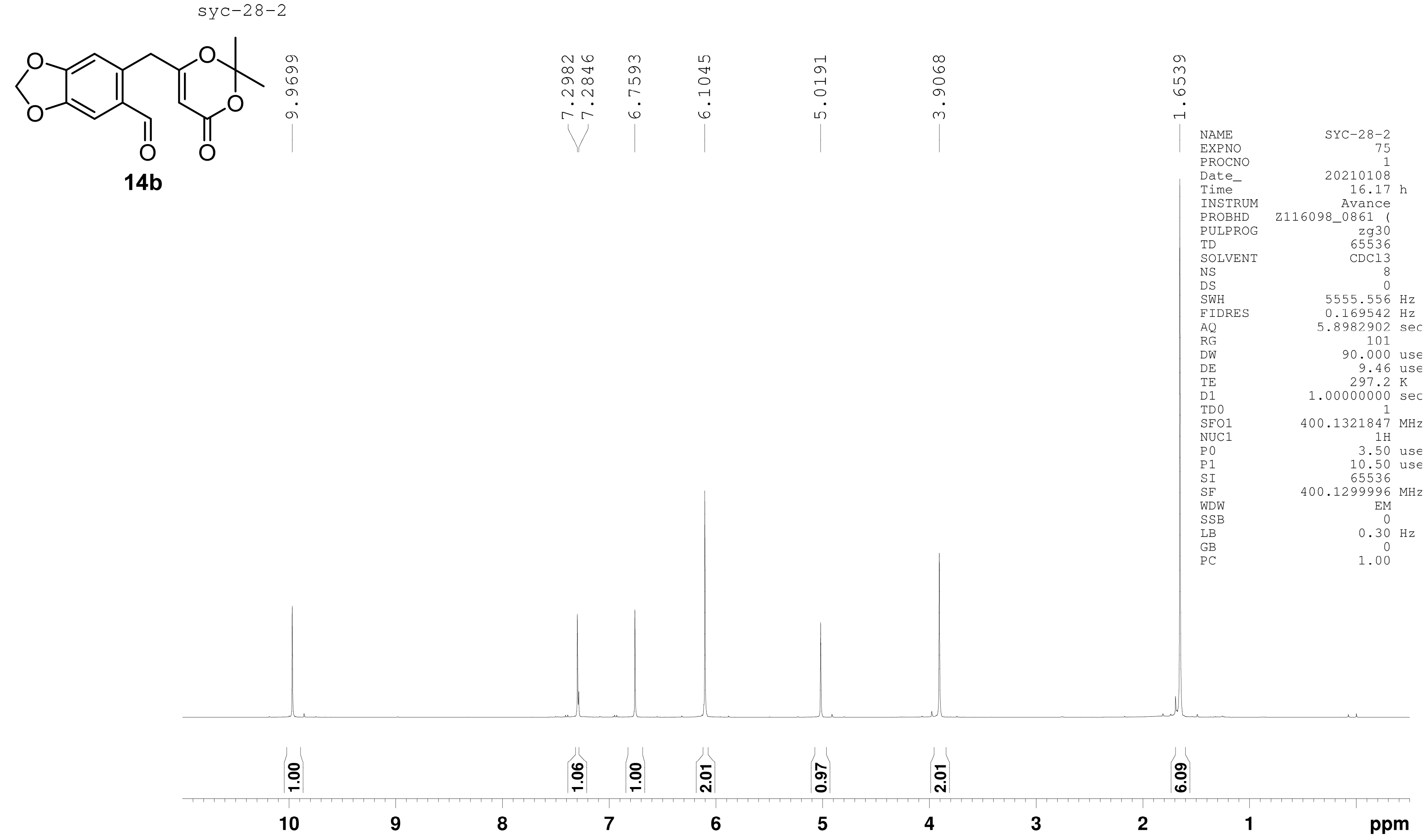

Figure S17 ^{1}H-NMR (400 MHz, $CDCl_3$) spectra of **14b**

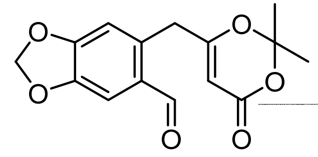

14b

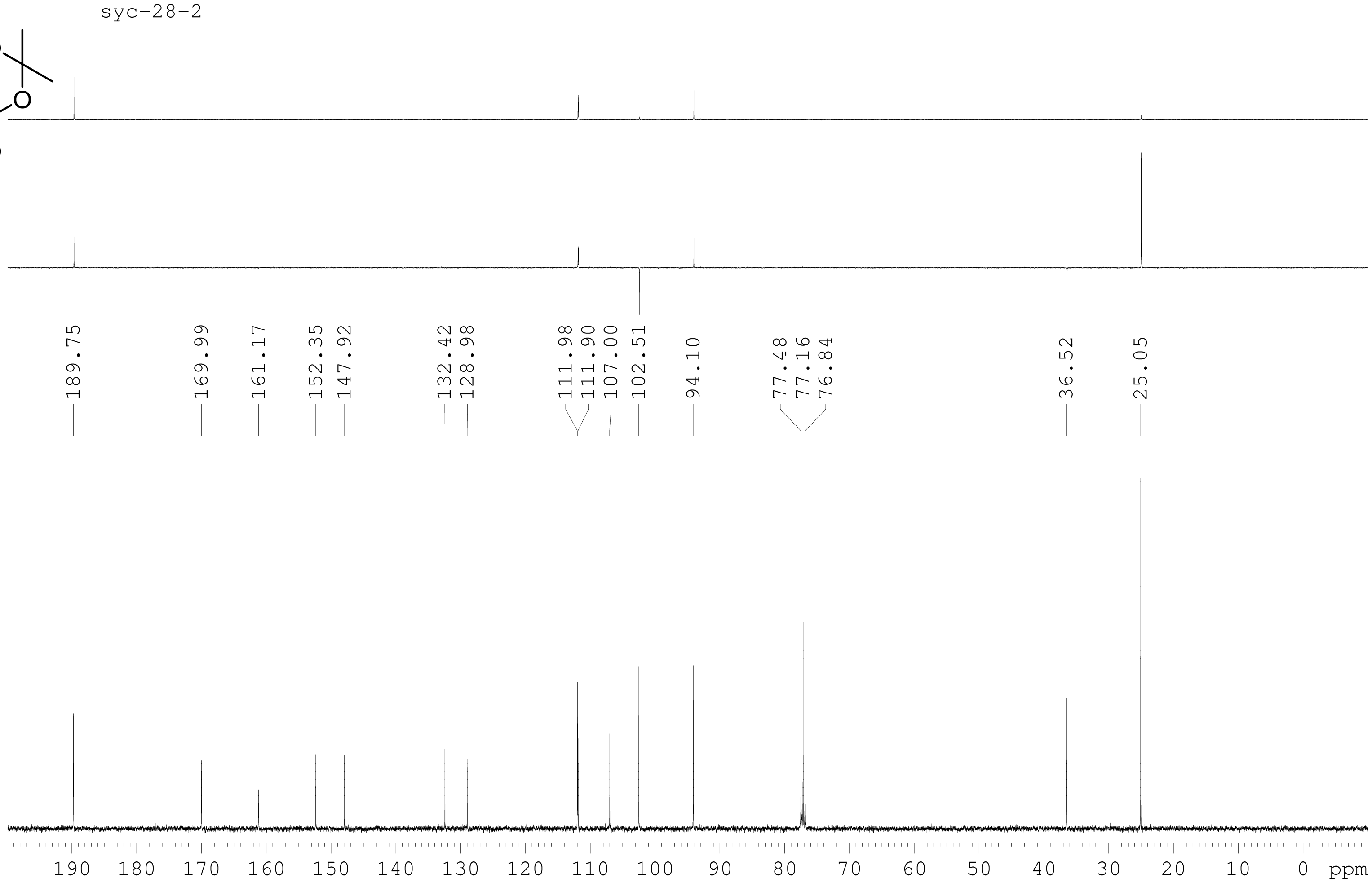

Figure S18 ^{13}C-NMR (100 MHz, $CDCl_3$) spectra of **14b**

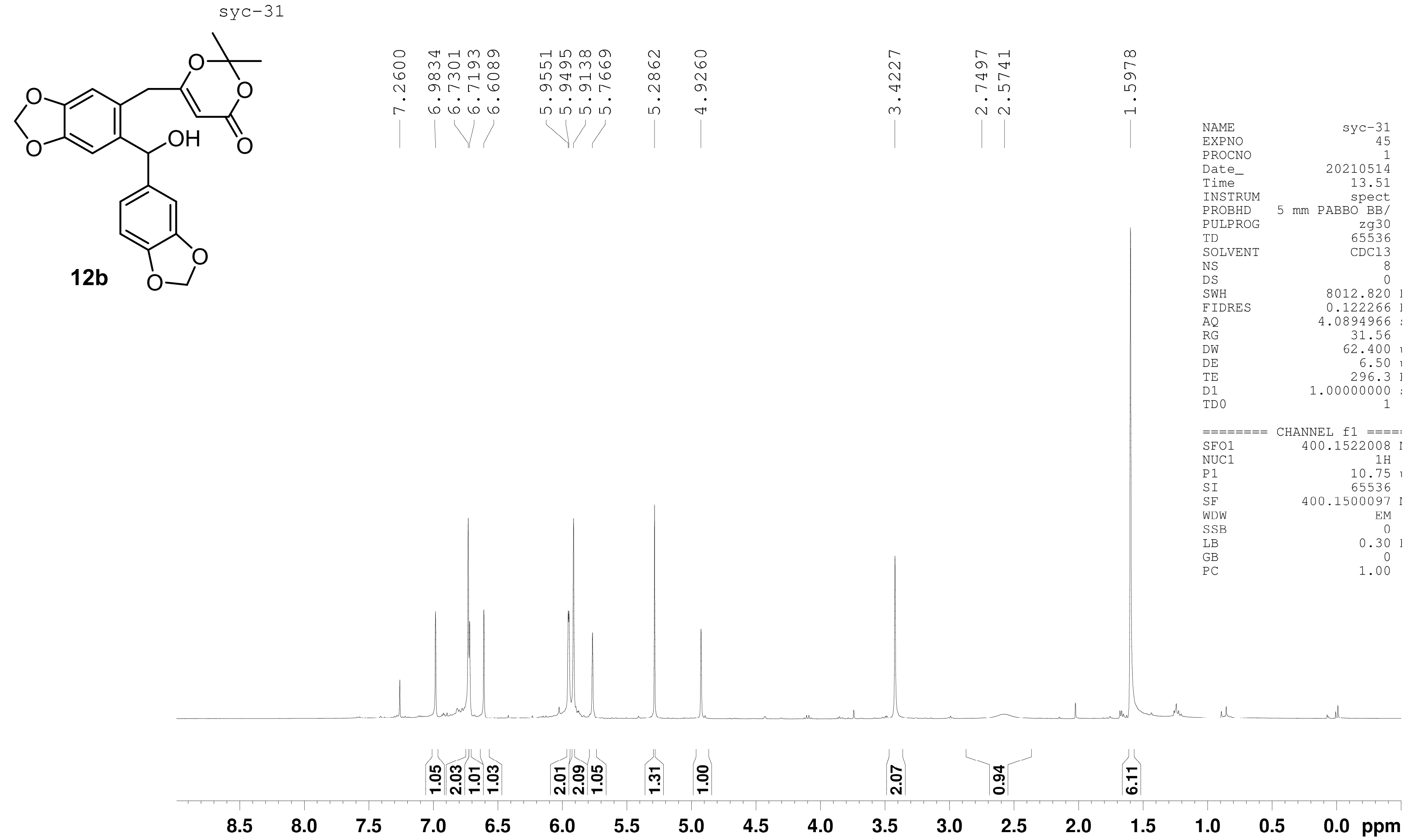

Figure S19 ^{1}H-NMR (400 MHz, $CDCl_3$) spectra of **12b**

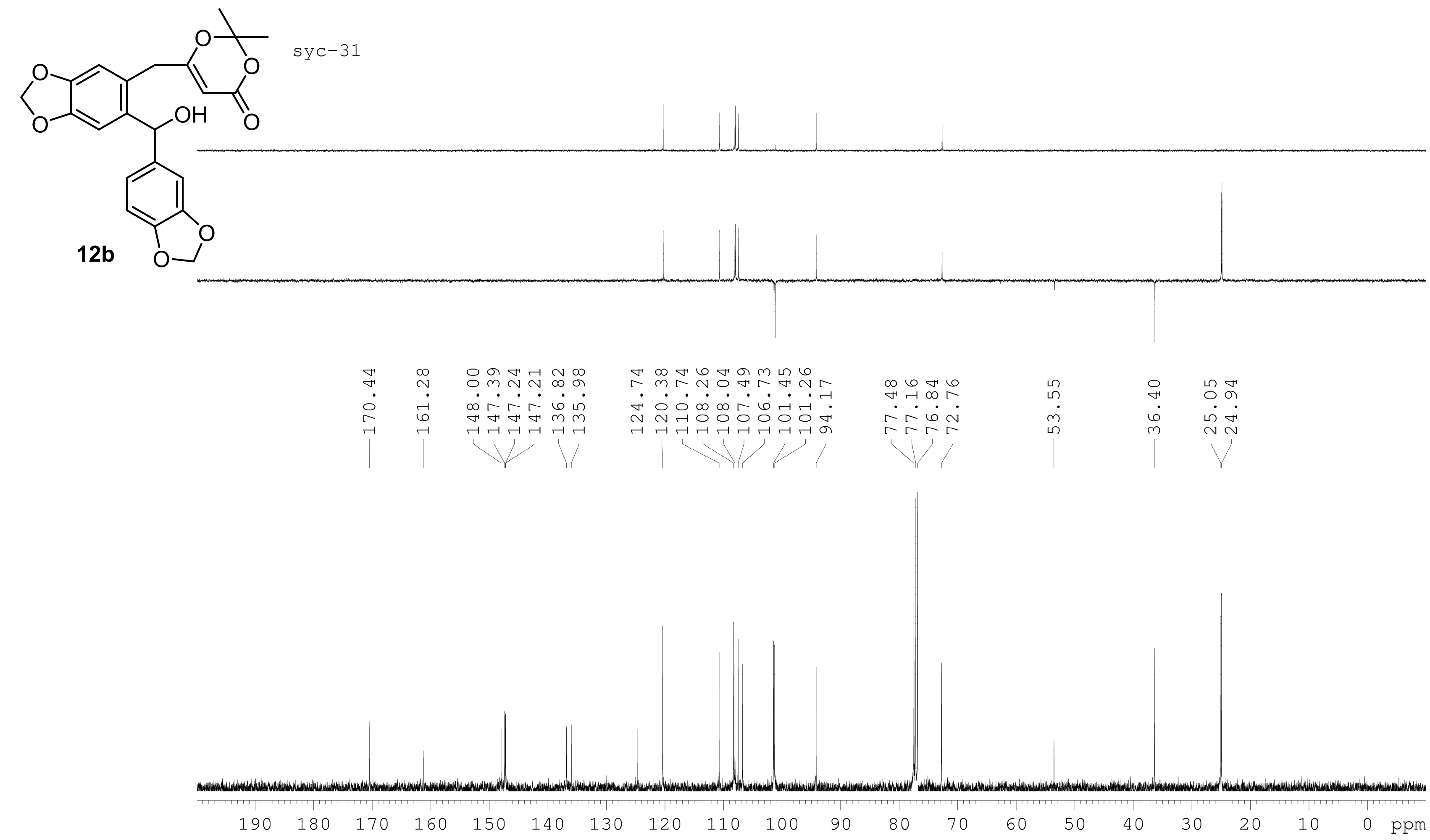

Figure S20 ^{13}C-NMR (100 MHz, $CDCl_3$) spectra of **12b**

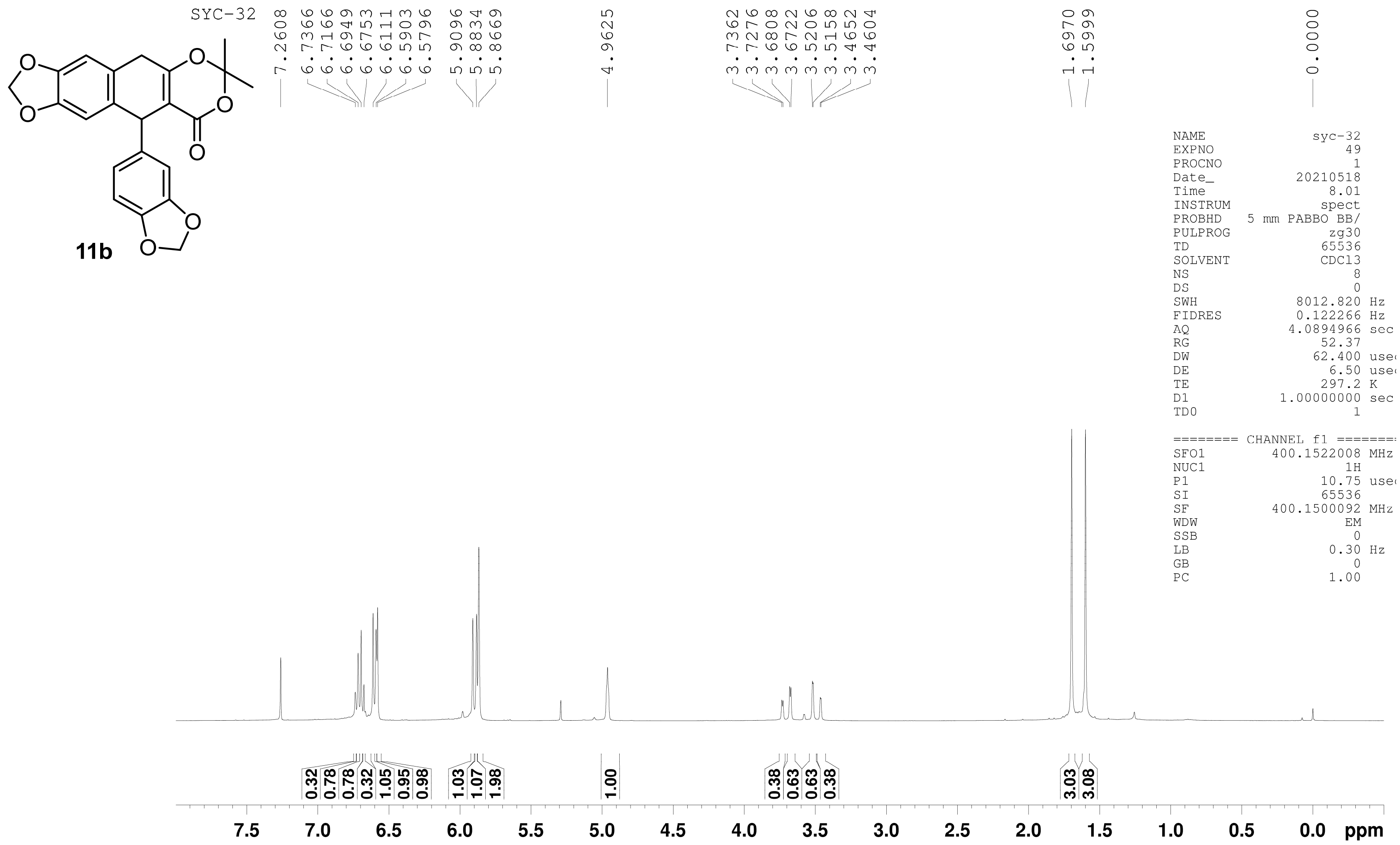

Figure S21 ^{1}H-NMR (400 MHz, $CDCl_3$) spectra of **11b**

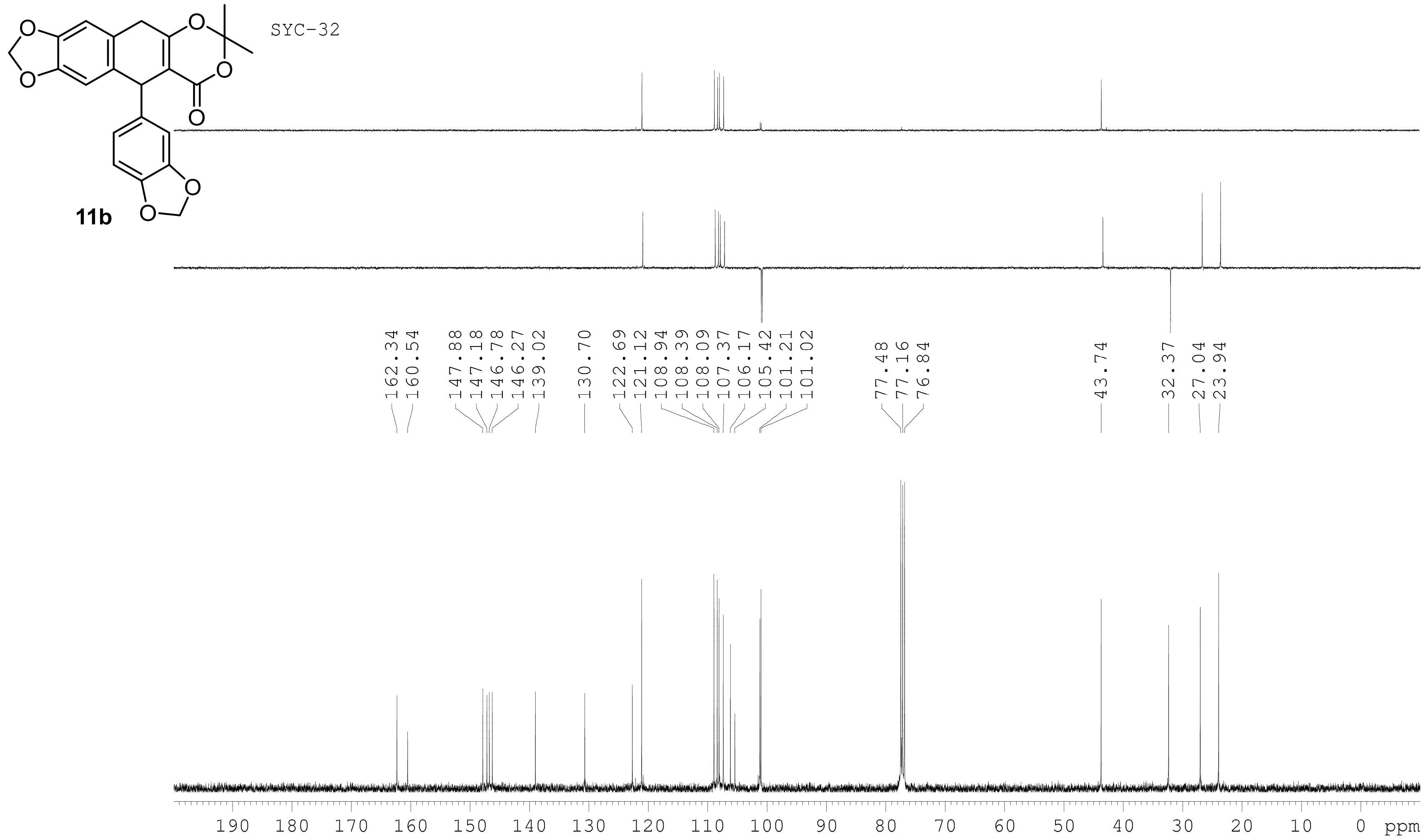

Figure S22 ^{13}C-NMR (100 MHz, $CDCl_3$) spectra of **11b**

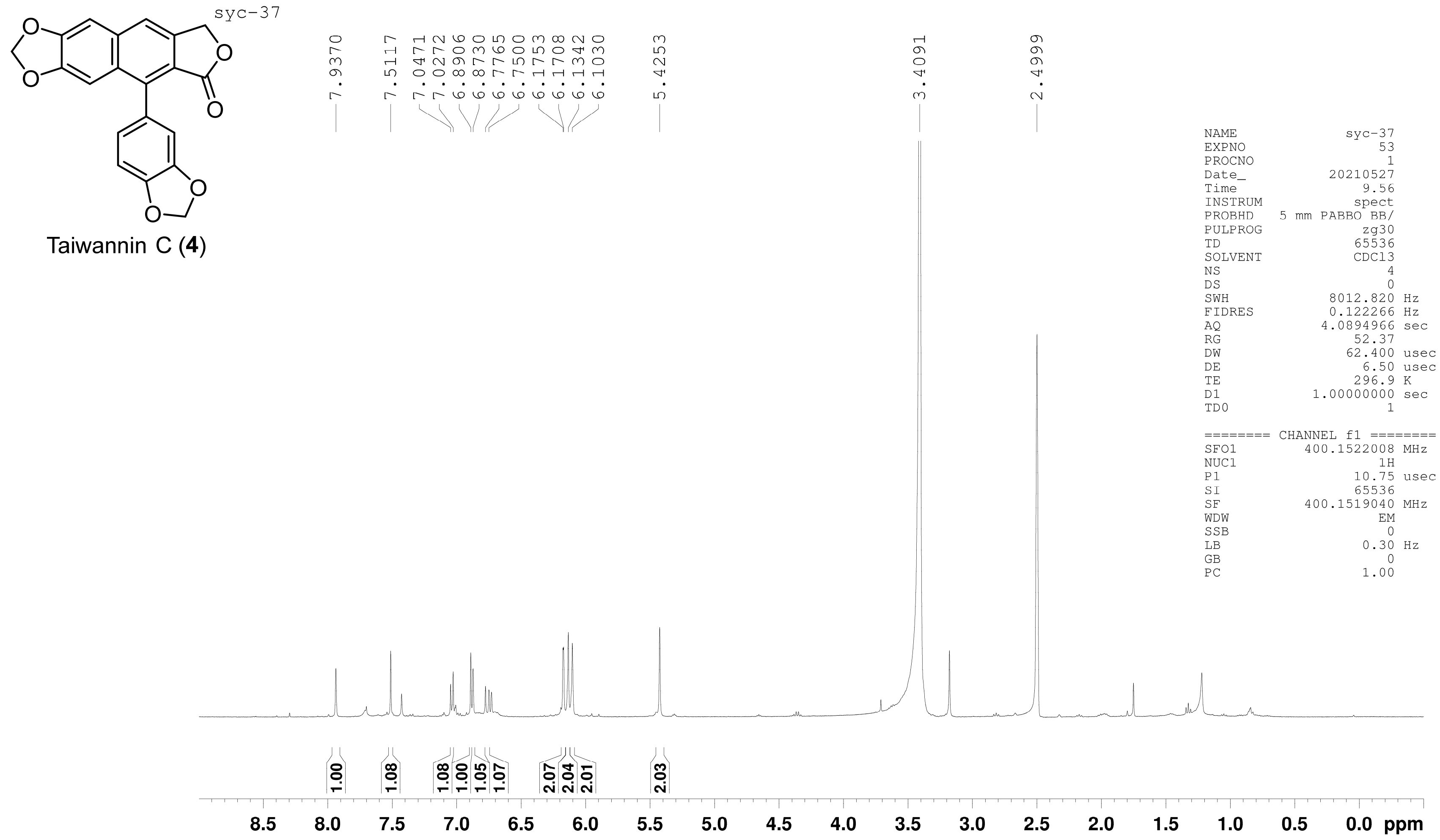

Figure S23 ^{1}H-NMR (400 MHz, DMSO) spectra of Taiwannin C (**4**)

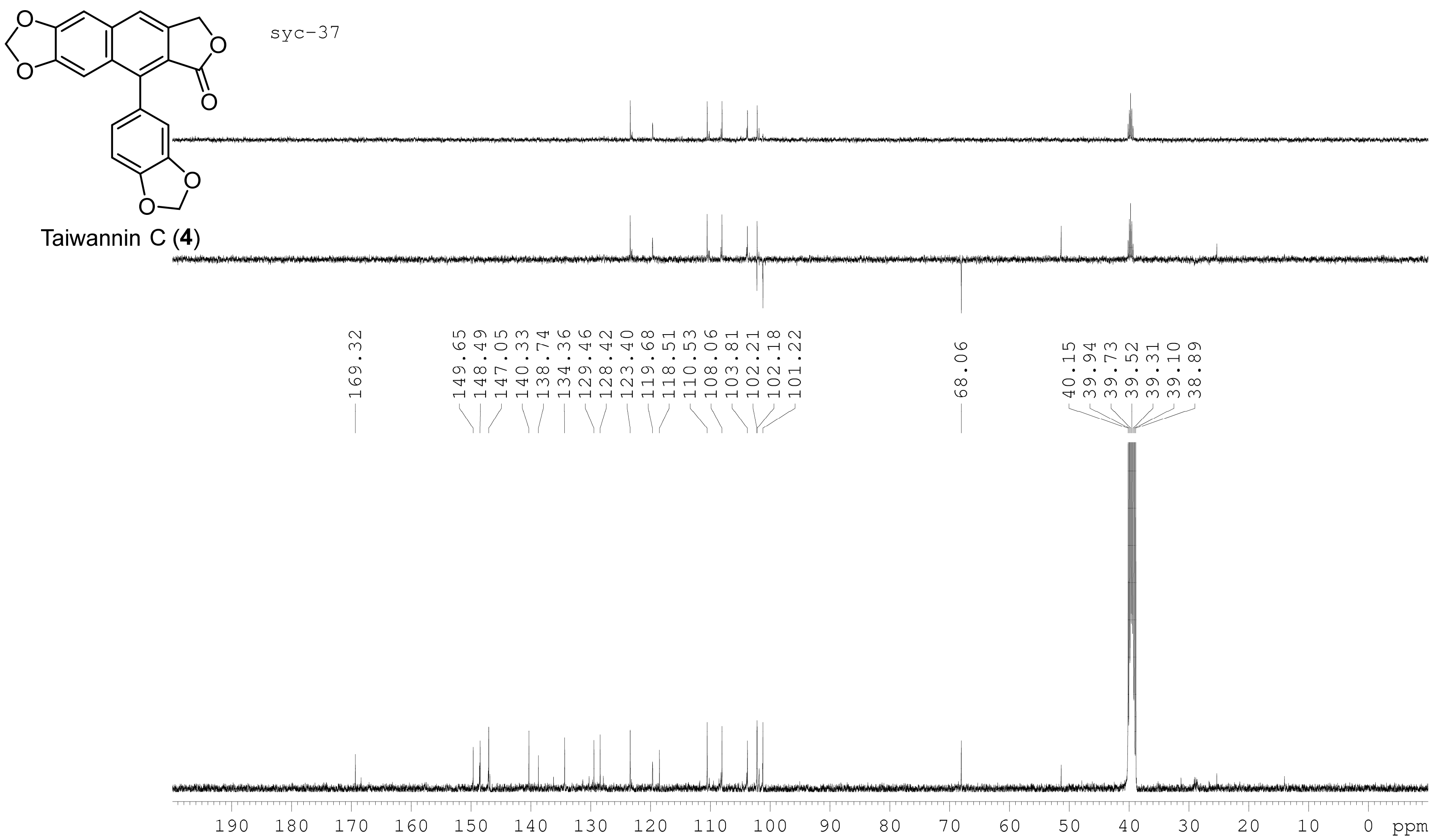

Figure S24 ^{13}C-NMR (100 MHz, DMSO) spectra of Taiwannin C (**4**)

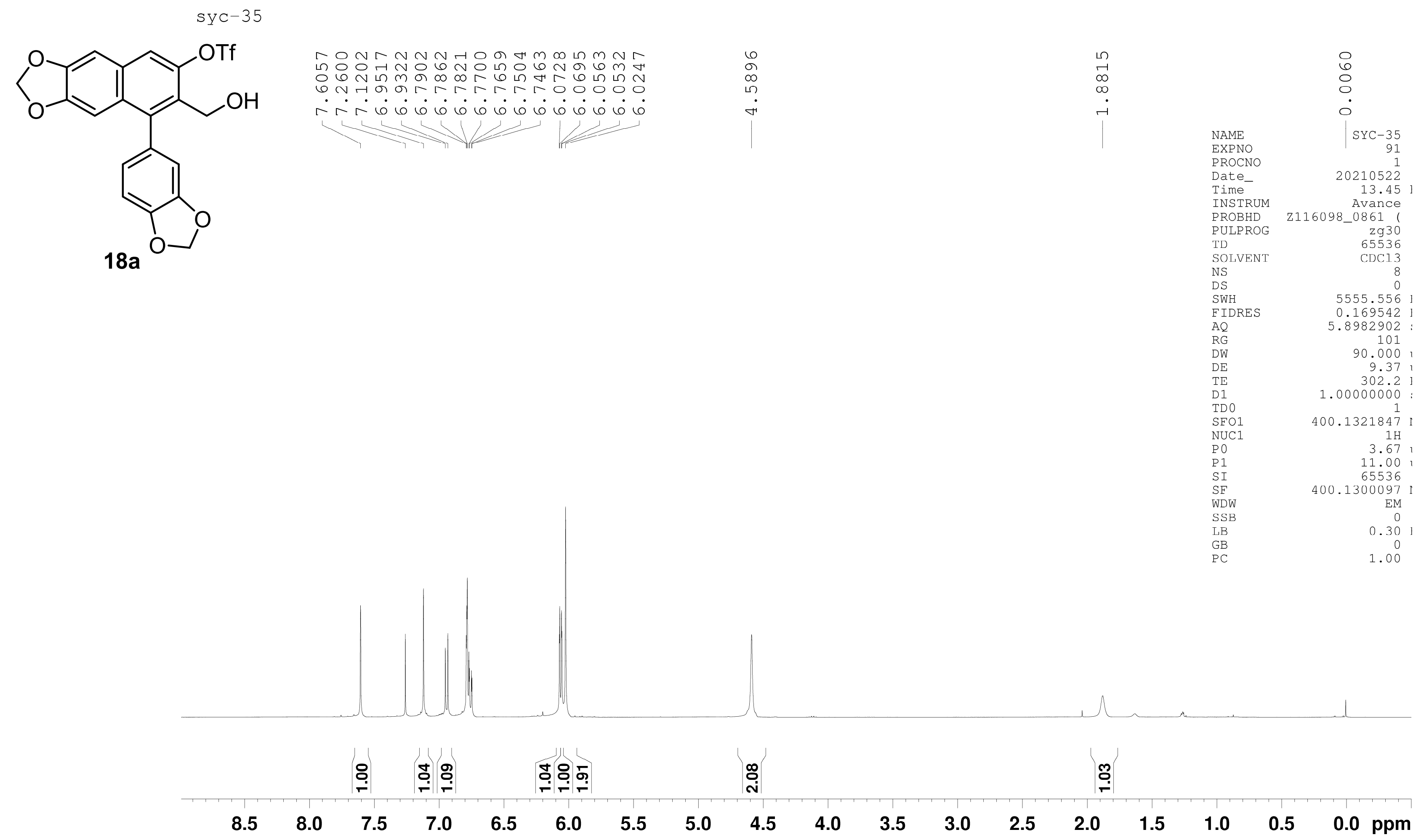

Figure S25 ^{1}H-NMR (400 MHz, $CDCl_3$) spectra of **18a**

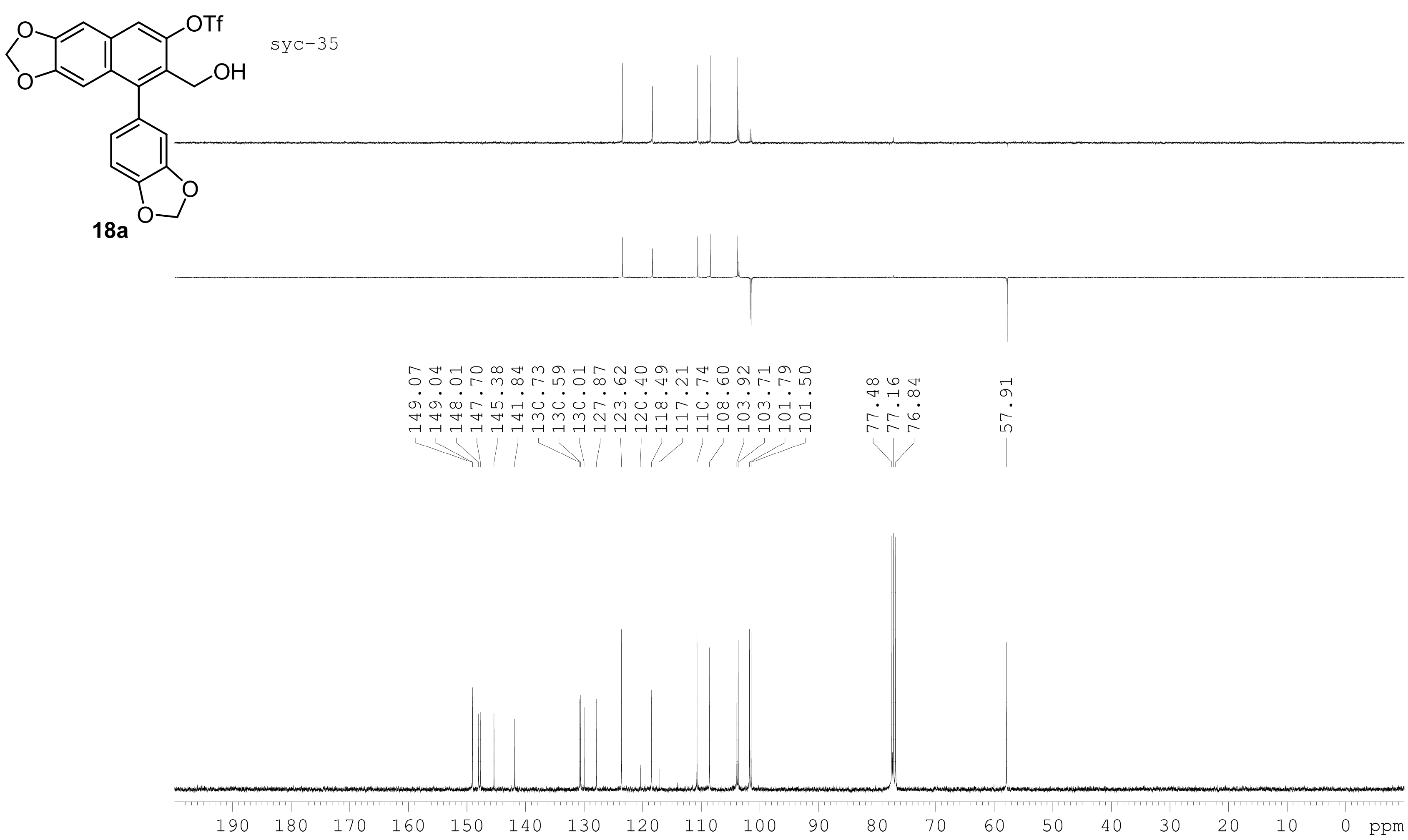

Figure S26 ^{13}C-NMR (100 MHz, $CDCl_3$) spectra of **18a**

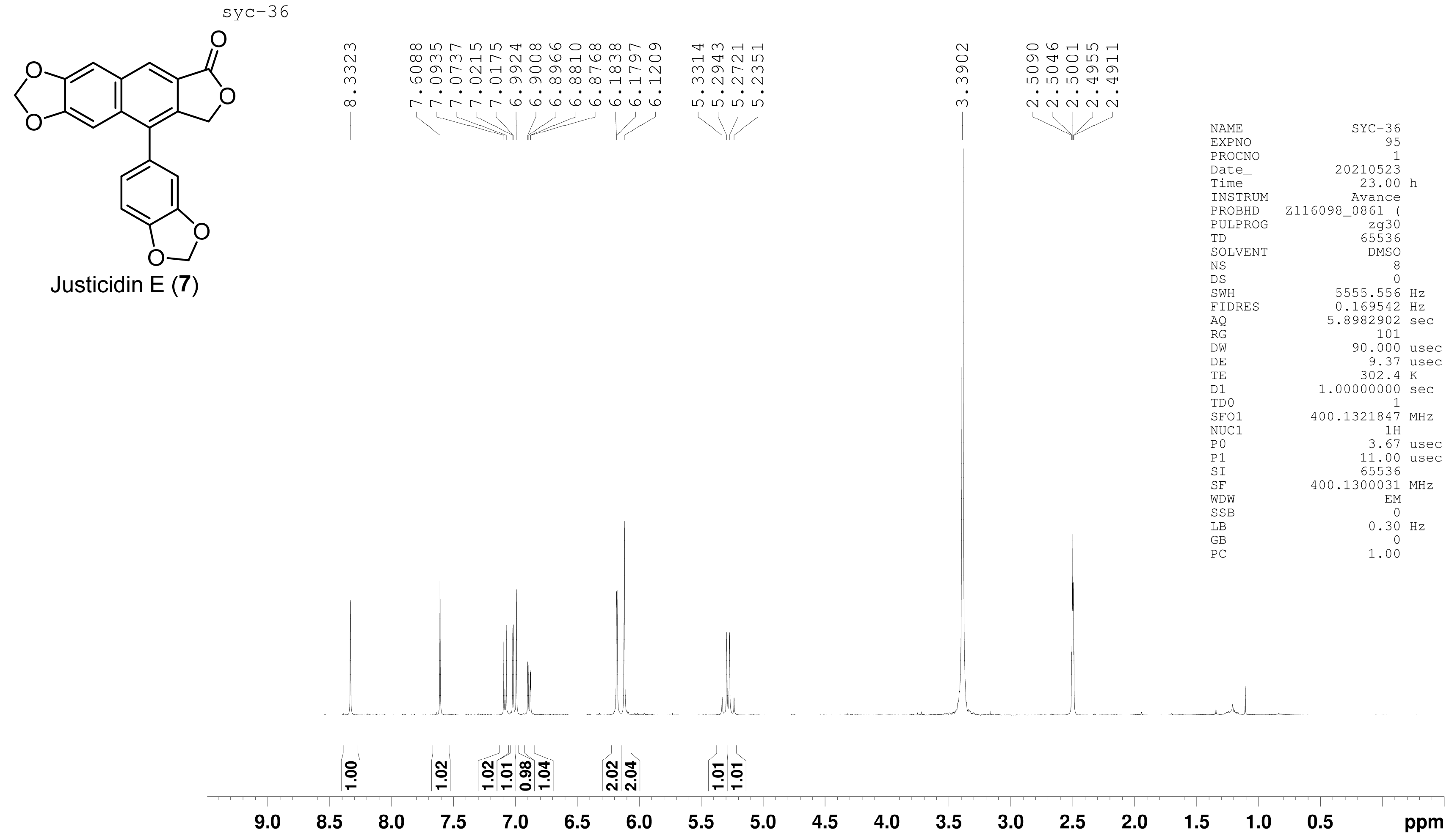

Figure S27 ^{1}H-NMR (400 MHz, DMSO) spectra of Justicidin E (**7**)

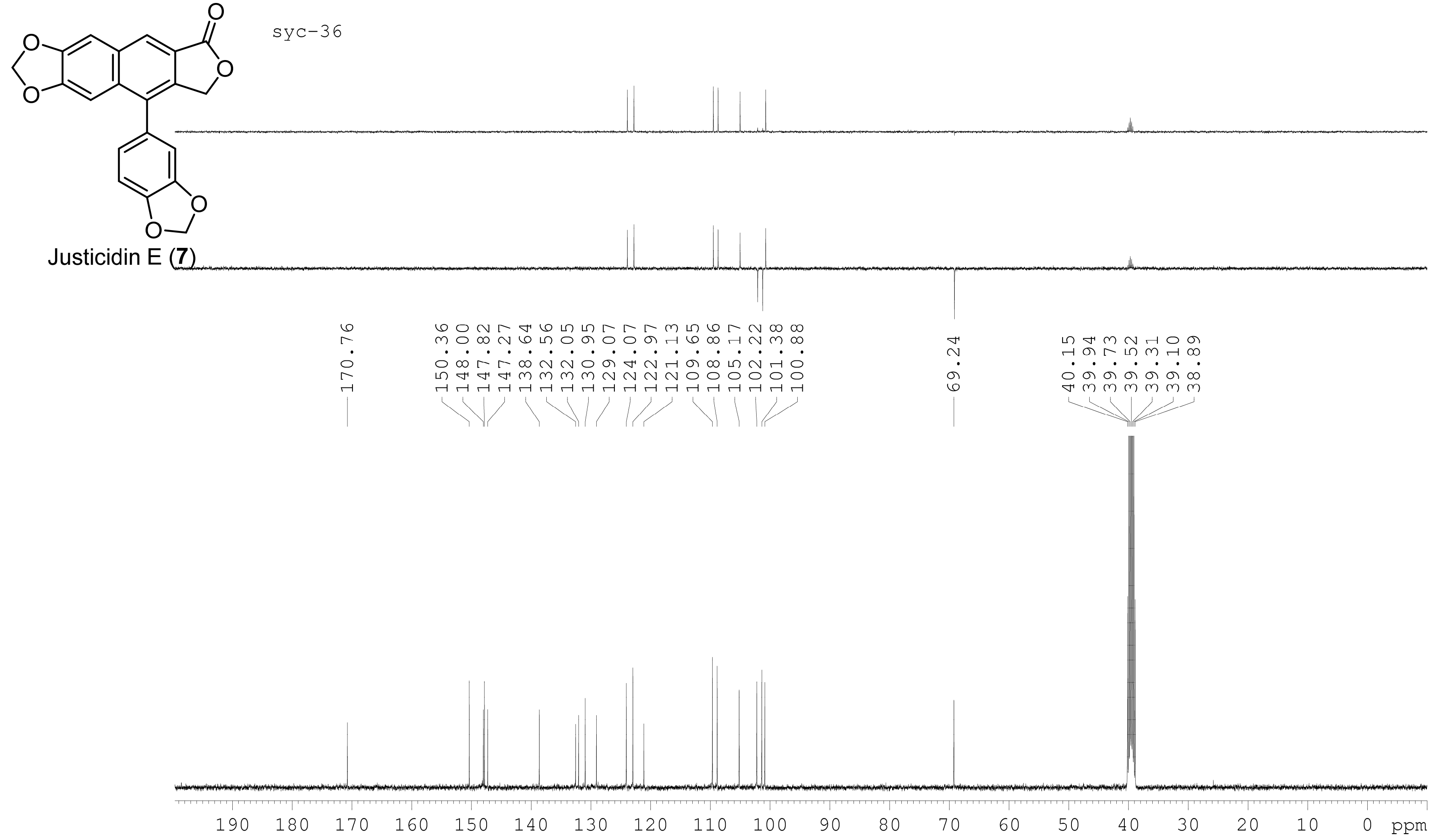

Figure S28 ^{13}C-NMR (100 MHz, DMSO) spectra of Justicidin E (**7**)
